# Stakeholders’ views and experiences of care and interventions for addressing frailty and pre-frailty: A meta-synthesis of qualitative evidence

**DOI:** 10.1371/journal.pone.0180127

**Published:** 2017-07-19

**Authors:** Barbara D’Avanzo, Rachel Shaw, Silvia Riva, Joao Apostolo, Elzbieta Bobrowicz-Campos, Donata Kurpas, Maria Bujnowska, Carol Holland

**Affiliations:** 1 Unit for Quality of Care and Rights Promotion in Mental Health, IRCCS Istituto di Ricerche Farmcologiche Mario Negri, Milan, Italy; 2 Aston Research Centre for Healthy Ageing, Aston University, Birmingham, United Kingdom; 3 Department of Oncology and Hematology, University of Milan, Milan, Italy; 4 Health Sciences Research Unit: Nursing, Nursing School of Coimbra, The Portugal Centre for Evidence-Based Practice: a Collaborating Centre of the Joanna Briggs Institute, Coimbra, Portugal; 5 Department of Family Medicine, Wroclaw Medical University, Wroclaw, Poland; Universita degli Studi di Firenze, ITALY

## Abstract

Frailty is a common condition in older age and is a public health concern which requires integrated care and involves different stakeholders. This meta-synthesis focuses on experiences, understanding, and attitudes towards screening, care, intervention and prevention for frailty across frail and healthy older persons, caregivers, health and social care practitioners. Studies published since 2001 were identified through search of electronic databases; 81 eligible papers were identified and read in full, and 45 papers were finally included and synthesized. The synthesis was conducted with a meta-ethnographic approach. We identified four key themes: *Uncertainty about malleability of frailty*; *Strategies to prevent or to respond to frailty; Capacity to care and person and family-centred service provision*; *Power and choice*. A bottom-up approach which emphasises and works in synchrony with frail older people's and their families' values, goals, resources and optimisation strategies is necessary. A greater employment of psychological skills, enhancing communication abilities and tools to overcome disempowering attitudes should inform care organisation, resulting in more efficient and satisfactory use of services. Public health communication about prevention and management of frailty should be founded on a paradigm of resilience, balanced acceptance, and coping. Addressing stakeholders’ views about the preventability of frailty was seen as a salient need.

## Introduction

### Objective

This meta-synthesis of qualitative studies examines stakeholders' understanding, beliefs and attitudes towards screening, intervention and care in the context of frailty and pre-frailty, in order to understand perceived or observed value for these healthcare activities, or experiences of individuals in relation to them. It aims to examine views across the range of involved stakeholders: frail and healthy older persons, informal carers, and health and social care practitioners.

This meta-synthesis is conducted in the framework of an EU funded project aimed at reducing the burden of frailty in the European Union, *Frailty management Optimisation though EIP AHA Commitments and Utilisation of Stakeholders input (FOCUS)*.

### Background

Frailty is defined as a state of high vulnerability to the risk of adverse events when exposed to a stressor [1], for example, a new diagnosis or exacerbation of a previously stable chronic condition, a fall or a traumatic life event. The frailty process appears to be a transitional state in the dynamic progression from robustness to functional decline [2], during which physiological reserves decrease and become less likely to be sufficient for maintenance or repair of the consequences of conditions. That is, frailty can be seen as the absence of resilience.

There are two main operational descriptions of frailty in the literature, which are both used to define frailty in clinical contexts. The first is a phenotype [3] which defines frailty using physical markers focused on muscle strength/weakness and sarcopenia. The second is that of an accumulation of deficits [4] which is flexible to context but, in addition to physical weakness, includes accumulated morbidities, polypharmacy, cognition, mental health and activities of daily living, although debate continues as to the range of approaches that are appropriate to detect frailty. Within these two frameworks, many tools have been developed to define a person as robust, pre-frail or frail [5,6,7]. The choice of tool can be determined by the purpose of the specific assessment, for example, directed towards an intervention that is to be provided based on screening. Prevalence of frailty in the population aged over 65 varies from 4 to 17% depending on definition and population, with 26% of over 84s being frail [8].

Development of the accumulation of deficits models has underlined the necessity to integrate medical with social domains to really understand frailty, recognising that frailty also defines a social group. Nicholson integrates the concepts of marginal groups and liminality between a possibly active and empowered group (the Third Age) and an invisible, totally dependent and near to death group (the Fourth Age) [9]. According to this theory, the defining feature of this Third Age/Fourth Age conceptualisation is that it constructs the possibility that individuals can be "in transition", without membership of either group. This liminality also represents the idea of fluid space where some form of power can be still experienced, at the same time as vulnerability and dependence [10]. While adding complexity to the view of frailty, the social conceptualisation offers a clear framework to the possibility of resilience and some form of power within frailty.

However we conceptualise it, frailty likely has a huge social impact. There are clear indications that frailty increases the risk of isolation [11], and therefore necessitates the commitment of services to address a difficult-to-reach group. Nevertheless, it is increasingly clear that frailty is malleable [12]. This understanding is timely. It relates to our context whereby European and worldwide healthcare systems are undergoing transformation efforts to move from single diagnosis focus, and systems where medical and social care function in separate silos, to more holistic care which considers the accumulation of effects on a patient and aims for an integrated approach [13].

Frail older people are high users of informal and formal care, and healthcare services [14]. However, studies illustrate examples of older people and their carers refusing services offered [15], or expressing reluctance to seek care even whilst acknowledging that such support is needed [16]. There are also indications that dependency on services, but limited participation in decision making, can produce feelings of devaluation and disempowerment in older persons [17]. It is essential to take into account older adults’ views, attitudes and understandings related to their own coping and care planning strategies, and to care and interventions in the context of frailty.

Informal caregivers receive support in many jurisdictions, but there is a perception that what support they get is based on fixed formulae regarding eligibility for fixed support, rather than their own perceptions of needs or what support would make a difference [18]. We wanted to integrate their points of view as well.

Previous studies on screening and intervention programmes in other health areas have found not only that potential patients are often reluctant to be screened or to follow health advice, but GPs and Practice Nurses (PNs) may be reluctant or unable to implement the intervention as was structured [19,20], suggesting lack of fit with understanding, perceived value, and skillset. An awareness of issues faced by practitioners in implementing care programmes or supporting care of frail elders, including their perceptions of the roles, needs integrating into the knowledge base in this area.

Qualitative research works to produce knowledge from subjective experiences. This can be both necessary and powerful when those points of view are from at risk and marginalised groups whose voices would otherwise not be heard. Frail older people are considered such a group. Qualitative research enables frail older people to express their needs and describe experiences in terms of what matters to them in their everyday lives. These data are then analysed systematically to represent the needs of older adults when evaluating and planning services. Widening the evidence-base in health and social care service development to include qualitative research is especially powerful when assessing acceptability and feasibility of interventions within particular population groups, services and communities [21].

The review teams consists of research centres across Europe with expertise in frailty, care for older people and disability related to mental disorders spanning the disciplines of psychology, health and social sciences, and medicine. We saw a need to review the qualitative evidence on this issue because there were questions about the experience of disability, well-being, frailty and health that were being obscured in more traditional reviews owing to their inherent relationship with services, institutions and power. Prioritising the voices of older adults and those with responsibilities towards them is commensurate with the review team's commitment to a recovery-oriented approach to care for older people that encompasses the concepts of resilience and empowerment in an authentic way.

We have conducted this meta-synthesis in order to build on the qualitative literature of frailty and reach a more encompassing vision of the issue. In doing this, three main ideas have orientated our work. These were: how much frailty is seen as malleable and preventable by stakeholders; the scope for integrated and empowering care; the importance of the knowledge and practices that stakeholders, frail older people in particular, use to cope with the conditions of frailty.

## Methods

### Design

We conducted a meta-synthesis of qualitative evidence because the objective was to make sense of the evidence base rather than simply collate it. An interpretative synthesis method was therefore appropriate [22–24] in order to understand the conceptualisation of frailty through the views, and attitudes of stakeholders. Such evidence is needed to inform the development of frailty interventions that are both feasible and acceptable within the context of health and social care in Europe.

### Search strategy

Three electronic databases were searched in July 2015: PubMed, CINAHL and Web of Science. The search strategy included terms relating to frail older people (population), frailty (phenomenon of interest), qualitative or nursing methodology research and mixed methods studies (methodology) (S1 Text; search strategy is shown for PubMed, with the same applied to CINHAL and Web of Science with appropriate modifications).

### Inclusion and exclusion criteria

Included studies were: qualitative research; with frail older adults or stakeholders involved in their care, e.g. nurses, allied health professionals, family caregivers; about the conceptualisation of frailty; beliefs about interventions to prevent or manage frailty. We selected qualitative research studies as identified through the MeSH terms and several appropriate keywords in the searched databases (S1 Text, Search strategy). The MeSH terms used in PubMed were "qualitative research", "nursing method research", "focus groups", "interviews as topic". These terms were integrated with keywords searched in title and abstract, including again terms related to the methodology (like "qualitative study" or "mixed methods", and others) and terms related to the topic (like "point of view", "perception", and similar). All the possible terms were used in MeSH and in title and abstract in order to assure identification of all the relevant papers from the searched databases.

We included studies published in the period 2001–2015 in English, French, Spanish, Portuguese, Italian and Polish, which were the languages that at least two researchers in the group could read. Studies were excluded if they did not have frailty as a focus, if they were conducted in specialised communities, e.g. homeless, if they focused on specific diseases, end of life care, neglect or abuse of older adults, or organizational interventions.

### Screening

Seven members of the review team (MB, BD, CH, DK, RN, SR, RS) screened records by title and abstract against inclusion criteria. All records were screened independently by two people to check for consistency; disagreements were resolved by discussion within the pair and consultation with another team member if required. Full text papers were further screened, each by two people, to make final decisions about inclusion.

We did not find any paper in Italian, Polish or Spanish. The papers in Portuguese could be read by the Potuguese (JA, EC), UK (RN) and Italian members (BD).

### Quality appraisal

Included papers were appraised using the Critical Appraisal Skills Programme checklist (CASP) for qualitative research (http://www.casp-uk.net/; S2 Text). The evaluators (JA, MB, EC, BD, CH, DK, RN, SR, RS) conducted the quality appraisal independently in pairs and any differences concerning inclusion were resolved by discussion within the pair or through consultation with another team member.

### Synthesis

The synthesis was informed by meta-ethnography [25–27]. We chose meta-ethnography because we aimed to not only aggregate findings from papers but also to develop a conceptual understanding of what the original studies say. This higher order, synthesised conceptualisation of the evidence base will be used to contribute to best practice guidelines for frailty management and prevention to be developed in the FOCUS Project [28,29]. In developing the synthesis, we referred to the seven phases described by Noblit and Hare [25,27,30]. After having formed specific research questions (Phase 1, *Getting started*) and defined the material through search strategy, acquisition and selection of papers, and quality appraisal (Phase 2, *Deciding what is relevant*), all papers were repeatedly read by the authors. This was a continuous process which lasted until the end of the synthesis (Phase 3, *Careful reading and re-reading*), enabling development of a good understanding of all papers. Following Toye’s (2014) [25] conceptualisation of first- and second-order constructs, we tabulated extracted data as first- and second-order constructs. We extracted constructs verbatim using the theme titles (or subheadings) used by authors. Where theme titles were absent we created descriptors to code the content. If we experienced difficulties in identifying an appropriate descriptor, we consulted the discussion section of papers to help consolidate the authors’ interpretations.

A grid of concepts was then developed (Phase 4, *Determining how the studies are related*), which served to prepare the reciprocal constant translation of studies. This process was shared with six team members (MB, BD, DK, CH, SR, RS) reviewing all theme clusters to ensure coherence and representation of all included studies. The large initial grid of concepts was refined in order to achieve a manageable and useful synthesis (Phase 5).

The lines-of-arguments synthesis (Phase 6) involved interpretative work to identify major themes, within the synthesized findings. The set of synthetic themes (third-order constructs) were derived from results of the reciprocal translational analysis and discussions within the team to reach consensus.

This interpretative phase of the synthesis was led by three members (BD, CH and RS) and agreed by the full team. The final set of themes was used to develop a conceptual model of frailty. This was achieved through discussion within the analytic team to arrive at a shared understanding of relationships between themes. The reasoning behind each connection was analysed. Themes were moved around into new positions to test out their meaning and inter-relationships. After varying the patterns, a model was developed which depicted a new, more consistent and integrated conceptualisation of frailty, taking into account multiple meanings, and perspectives from different stakeholders.

First-order constructs can be considered in the middle between the raw data and second-order constructs, or even second-order constructs themselves. First-order constructs identified and selected by the authors according to their second-order constructs (Toye et al., 2014) [25] are presented in the results of the synthesis. When these were not given, or given to a very limited extent, we explained the second-order constructs without first-order extracts.

## Results

### Included studies

**Fig 1:**
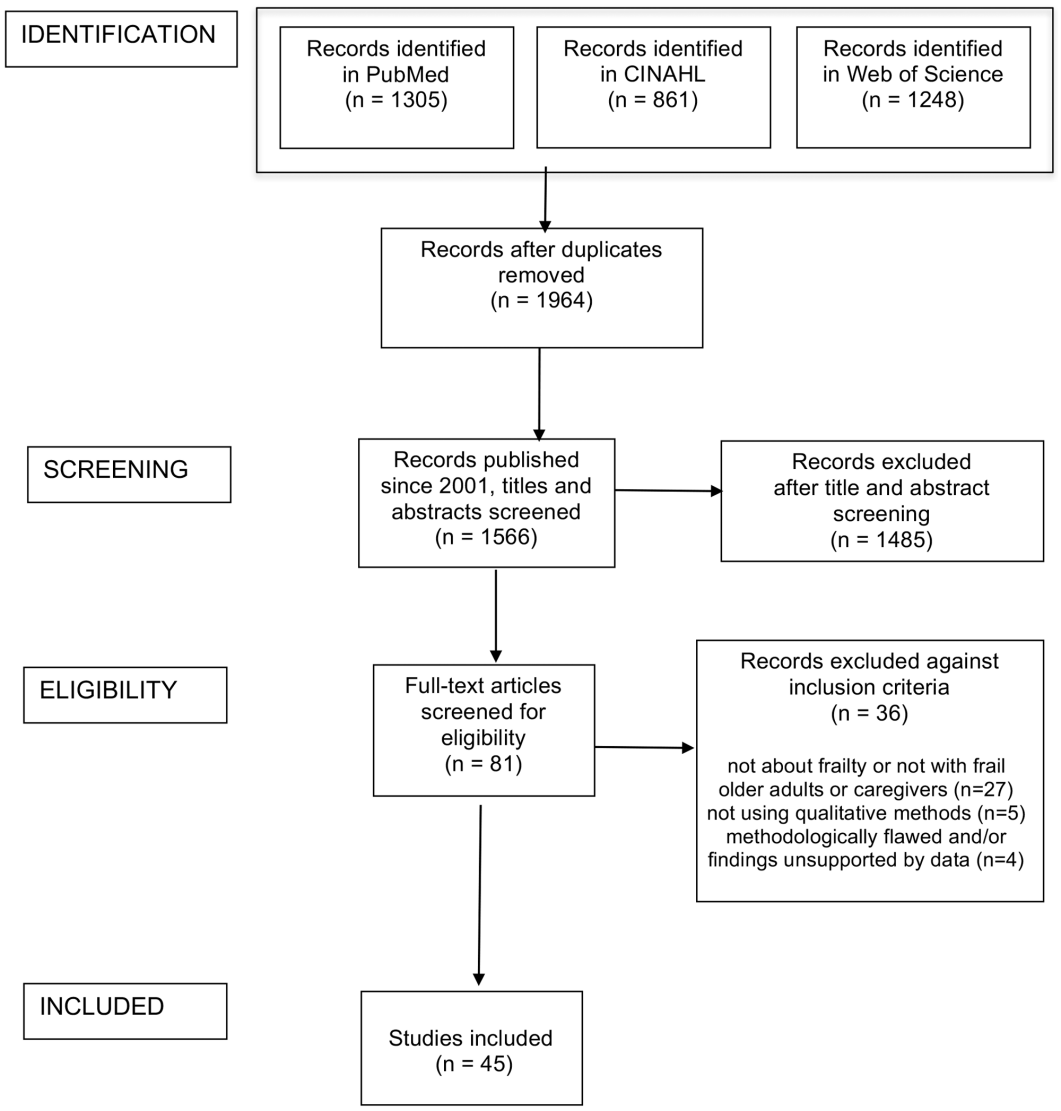
PRISMA flow diagram.

A total of 3414 records were identified as potentially relevant and this number was reduced to 1964 after removal of duplicates. Limiting the papers to those published since 2001 resulted in a total of 1566 papers. Titles and abstracts of these papers were read to identify the relevant papers and this process led to the exclusion of 1485 papers. Eighty-one papers were read in full text and appraised for quality. Among these, 36 were excluded: 27 did not address frailty or did not include frail older people or their caregivers and professionals (including seven studies addressing implementation and organization issues); five used methodologies other than qualitative (including one sociological study using sociological categories and conceptualizations); four were very poor quality (did not give information about analysis, gave very limited information about methods, or presentation of findings was not supported by the data) (Fig 1). S1 Table illustrates the main chracteristics of the 45 included studies [9,31–74].

Quality appraisal revealed that none was judged as poor. The majority had clear statements of research aims, employed qualitative methods appropriately, had clear conclusions and were judged to make a valuable contribution to the evidence base. Studies performed less well in their reflections about potential impact of relationship between researchers and participants, and lacked full consideration of ethical concerns.

The majority of included studies were conducted in a limited number of Western countries, with a focus on Northern Europe. Most studies recruited frail older adults as their participants (n = 28) either solely or with another stakeholder group; ten papers included informal caregivers. The largest practitioner group was nurses (n = 7), followed by social workers (n = 3), occupational therapists (n = 2), GPs (n = 1), and physiotherapists (n = 1). Only one paper recruited non-frail older adults in terms of examining their beliefs about frailty and frailty prevention. Six papers included more than one stakeholder group, and three included more than two groups. Eleven studies used focus groups, either as the only method or in combination with individual interviews or questionnaire administration, and the others used individual interviews. In one case, interviews were done in pairs of caregiver and professional matched as caring the same frail older person [47]. The most used method of analysis was grounded theory (15 papers), then content analysis (10 papers), phenomenology (four papers), and thematic analysis (three papers), with the remaining papers using other methods.

### Synthesis

The synthesis produced four synthetic themes (third-order constructs): *Uncertainty about malleability of frailty*; *Strategies to prevent or to respond to frailty*; *The capacity to care and person and family-centred service provision*; *Power and choice*. Each theme is presented with example quotations from study participants. The contribution of each paper to the constructs is displayed in Table 1.

**Table 1:** Contribution of included studies to themes.

Reference	Uncertainty about malleability of frailty	Strategies to prevent or to respond to frailty	The capacity to care and person/family-centred service provision	Power and choice
Ayalon L, et al. (2008) [31]			x	
Baillie L, et al. (2014) [32]			x	
Bindels J, et al. (2013) [33]	x		x	
Bindels J, et al. (2014) [34]			x	
Blanton PW (2013) [35]			x	
Bleijenberg N, et al. (2013) [36]	x		x	
Claassens L, et al. (2014) [37]		x		
Denson LA, et al. (2013) [38]			x	
Dick K, Frazier SC (2006) [39]	x		x	
Donlan WT (2011) [40]			x	x
Ebrahimi Z, et al. (2012) [41]		x		x
Ebrahimi Z, et al. (2013) [42]		x		
Ekelund C, et al. (2014) [43]			x	x
Ekwall A, et al. (2012) [44]		x		x
Faes MC, et al. (2010) [45]	x			
Fjelltun AM, et al. (2009) [46]			x	
Fjelltun AS, et al. (2009) [47]			x	
Grenier A, Hanley J. (2007) [48]		x		x
Gustafsson, S., et al. (2012) [49]	x			x
Hjaltadottir I, Gustafsdottir M (2007) [50]	x	x		
Horder HM, et al. (2013) [51]		x		
Kita M, Ito K (2013) [52]		x	x	
Koenig TL (2005) [53]			x	x
Kristensson J, et al. (2010) [54]			x	x
Levesque L, et al. (2010) [55]			x	
Lindhardt T, et al. (2008) [56]			x	
McGeorge SJ (2011) [57]			x	
Modig S, et al. (2012) [58]			x	
Nicholson C, et al. (2012) [9]		x		
Nicholson C, et al. (2013) [59]		x		
Puts MT, et al. (2007) [60]		x	x	
Puts MTE, et al. (2009) [61]	x			
Robben S, et al. (2012) [62]			x	
Roland KP, et al. (2011) [63]	x	x		
Rush KL, et al. (2013) [64]		x		
Sarvimäki A, Stenbock-Hult B (2014) [65]			x	x
Skymne C, et al. (2012) [66]		x		
Stockwell-Smith G, et al. (2010) [67]				x
Teixeira IN (2008) [68]	x			
Themessl-Huber M, et al. (2007) [69]		x		
Tutton EMM (2005) [70]			x	x
van Kempen JA, et al. (2012) [71]			x	
Walker R, et al. (2015) [72]			x	
Wallin M, et al (2008) [73]		x		
Zidén L, et al. (2008) [74]			x	

#### Uncertainty about the malleability of frailty

This theme was informed by nine studies [33,36,39,45,49,50,61,63,68] and includes examples of experiences and opinions which challenge or support the notion that frailty development is malleable. While health professionals showed confidence in prevention and management of frailty, they also acknowledged the difficulty of having the right tools to identify people who might benefit, and when intervention would be more useful. This worry also encompassed the belief that after a certain point frailty is not malleable anymore. The older adults had more uncertainties and thought frailty was, at least in part, inevitable and inherent to old age. Some also thought that frailty was the outcome of lifestyles and attitudes throughout life, and that prevention could hardly modify such long term impacts.

According to healthcare professionals, frailty was conceived as something which could be prevented through appropriate screening to detect pre-frailty, communication and timely pro-active interventions [33,49]. However, there was concern that current screening tools are inadequate and do not always target the right people: *“I don’t think people who are really frail fill in the form*. *It’s a big weakness”* [33]. Nonetheless, there was mixed response to interventions. Some studies showed older adults’ disbelief of the potential success of frailty interventions: *“They can’t take my fear of falling away”* [45]. Caregivers were also unconvinced: *“Because of her memory problems*, *she is not teachable anymore*.*”* [45].

Despite success with interventions such as physical therapy to reduce frailty [50], there was doubt elsewhere that any intervention with those living with co-morbidities was worthwhile: *“She is already 80 years old*. *With all her medical problems*, *such a programme would be useless*.*”* [45].

The understanding of the malleability of frailty was particularly clear in responses around the topic of the timeliness of interventions. While therapists noted that it was easier to identify frailty when people were significantly frail, they felt that it was too late to reverse at that point, but believed that the non-frail to pre-frail end of the spectrum was much more amenable to intervention. Although they were confident that their interventions could prevent frailty in these clients, they reported difficulty in working with them because they did not perceive themselves as at-risk. Thus, frailty screening needs to be proactive. However, among healthcare professionals the view that frailty could go past the point of no return was expressed: *“We’re always dealing with the consequences*, *by the time we get to it*, *it is too late*.*”* (physiotherapist) [63, p. 11].

Older adults often clearly stated that frailty was something inevitable although it is malleable if the person tries to stay in good health: *"Frailty is not something that you can prevent*, *you cannot do anything*, *it just happens when you get older*.*"* (frail respondent) [61, p. 264]

Some others saw inactivity as a cause of becoming frail, therefore indicating activity as a means to prevent frailty:

"… I see people they got married, got a garden in this complex and after that they are just sitting there all their life and doing absolutely nothing with their life..[…] You need to be active."

(non-frail respondent) [61, p. 263]

#### Strategies to prevent or respond to frailty

Here we present examples coming from sixteen papers, of how people aim to keep frailty at a distance or control it, for example, using strategies and behaviour to help cope with conditions which cannot be changed in themselves, changing their relationship with their conditions, indicating resilience [9,37,41,42,44,48,50,51,52,59,60,63,64,66,69,73].

Strategies include adaptive coping such as optimisation, maintaining social connections, and psychological and physical strategies to prevent or manage frailty. In terms of use of care this could include referral to services, with an emphasis on preference for services that would enable them to maintain their independence and ‘keep frailty at a distance’ [51].

Older adults gave examples of the development of personal strategies and their aims for these strategies to become habitual and overcome factors that impeded their efforts: “*live according to a certain rhythm*. *And then…I believe that*, *in due time*, *it will become automatic*.*… Be consistent*. *It's all about being consistent*.*”* (frail older adult) [37, p. 165]

Some studies reported clear cases of adaptive responses and psychological resilience in frail older adults. In one study, even very frail older adults were able to execute control by making their own decisions about when to seek support:

“…just thinking in a practical way about what I need. Something needs to be done, and I must try to do it…At a certain moment you make a sort of plan, and I should follow up on it.”

(85 year old woman) [37, p.163]

This adaptive coping illustrates older adults’ capacity for self-care, maintenance of social roles, sometimes assuming new roles in connecting to others, illustrating continuous growth throughout life:

"Well, that's difficult for me [working in the garden] so I'm now training a whole lot of junior gardeners. You'll be surprised, I'm training Jackie and anybody that comes now who's not a gardener; I'm training a whole new generation, because you see I have to remember every day."

(older participant) [59, p. 1177]

Being frail was described as requiring the mobilisation of resources to create well-being [41], responding to the imbalance imposed by frailty with a compensatory approach, and still focusing on selected personally important goals:

“If you learn to accept things as they are, use the time that otherwise would be empty and listen to tapes, then you can go on learning until you die.”

(frail older woman) [50, p. 52]

Mobilising resources also included the benefits of participation with other people and society:

"It's a matter of luck that I can still do so much, that my mind is still clear, that I can say something, that I can talk to them [caregivers].… I can chat with my children about the weather, about what's going on in the world… that's what I also talk about..… I can take part in things, to a certain extent."

(96 year old participant) [37, p.165]

Adaptive responses can assume the form of affirming “health despite frailty” [42] with evidence of maintained perceived balance and resilience in the context of physical impacts:

“There is nothing really wrong with my health, but then I get all these ailments. I have fallen five times and broken my arms and my leg and got a hip replacement and broken pelvis and all my ribs, I’ve broken everything, in fact. So that’s how I am, my bones are a chaos, but I have no health problems just now.”

(frail elder) [44, p. 15]

Acceptance of new conditions and devices to cope with them was also key to coping and maintaining their everyday life as much as possible:

"To dare to get up and go over to the toilet bowl, to this handicapped chair, it is an important part of health for me: There is mobility. To be able to get up and go even if it is with a cane, it is mobility."

(67-year old participant) [41, p. 1517]

Indeed, maintaining one’s routine was an explicit strategy to keep harmony and balance in everyday life:

"Health for me is that I feel well and I manage to do something I am used to doing. I have quite a routine situation, with no major changes.… I try to be active, have mobility, and keep up as much as possible."

(82-year old participant) [41, p. 1517]

In contrast, breaking the routine can be a way of falling into inactivity and losing connections to life:

"I’d love to stay like this [in her chair where she felt most comfortable]—you’ve only got to start doing that. You see my friend; she started staying in the chair. And then it was staying in her dressing gown and she seemed to just drop away then; that I sort of think to myself ‘no, you mustn’t do it; you’ve really got to keep on’. So I suppose I will, I’ll just keep on as long as I can."

(older participant) [9, p. 1429]

One study reported strategies used by older adults to resist organisational practices such as rationing care to those in crisis: *“I might refer to this as playing the frail old lady card…it’s sort of reverse denial*.*”* (frail older woman) [48, p. 220]. This strategy shows that the person knows how the system works and how she can obtain what she needs. In the face of health and social care systems which conserve their resources, this was a useful strategy.

Thus, keeping frailty at a distance means resisting the devastating impact frailty can have on one’s sense of integrity and identity, the possibility to see continuity between the past and present self [44]. Maintaining routine [9] and everyday life in familiar surroundings [42] is emphasised by these frail people, and having connections with family [50] and with the external world contribute to preserving identity, while accepting the changing body and circumstances [44].

These findings demonstrate that compensations and new balances, and even continued growth, are possible in face of physical, social and psychological changes.

#### The capacity to care and person and family-centred service provision

Twentysix papers contributed to this theme [31–36,38–40,43,46,47,52–58,60,62,65,70–72,74]. This theme presents findings related to care providers’ capacity to care, taking a patient-centred and family-centred approach to care. The significance of respectful relationships between carers and older adults, and between informal and formal carers, and their communication quality, were considered significant to care provision by different stakeholders. Some studies identified the roles of case managers or multidisciplinary teams as a way to overcome fragmentation or maintaining continuity of care [32,39,54,56,57].

A need for person- or family-centred approaches to care was a salient theme highlighted by the different stakeholder groups, who wanted to feel included in decision-making: *"I have felt redundant*, *that I cannot follow other people anymore*, *because I can’t see*. *I feel that they talk between themselves and I become excluded"* (frail elder) [65, p. 7]. Person-centredness was also reflected in the adaptability of services and systems to the individual’s needs, with findings that recipients of services found the inflexibility of services to be one reason why offers of support may be declined. More flexible use of respite care including shorter stays and enabling relatives to stay overnight was one example [40,46].

One recommendation for provision of family-centred care was to implement home visits that could foster the development of a partnership between care providers, caregivers and older adults, giving patients the opportunity to discuss their needs in a way that a home visit is more than just a health check or assessment, and focuses on what is needed to enable independence [33,71].

However, when different stakeholders' values were directly compared [38], there were still differences in perspectives regarding what constitutes care and what should be prioritised: frail older people prioritized autonomy and protection; family members prioritised safety, finances, and the value of living at home; and health professionals prioritised safety, autonomy and personal care. Older adults’ focus on autonomy was also evident in studies examining decision-making in care. Many expressed a desire to be actively involved in decision-making with nurses acting as facilitators, following a person-centred model [69,70].

Tutton (2005) [70] found older adults’ experiences of care were much more positive if they were able to develop a respectful relationship with care staff, if staff were friendly, gave them everyday choices, e.g. what to wear, where to sit, and provided care that was appropriate to their bodily abilities.

Time constraints were a reported threat to the implementation of family-centred services, though nurses were reluctant to use time as a valid excuse:

“Once in a while it is lack of time. Though I think it sounds a bit crude, too, to say that we haven’t got time, but sometimes it is [..] no, not time: we are not geared to talk to them in that way.”

(nurse) [56, p. 674].

This suggests that adopting a facilitator role and working collaboratively with older adults and family members to come to a joint decision does not fit with how they were trained to deliver care. Communicating in a more culturally sensitive and person-centred way [41,43], engaging in a ‘moral dialogue’ which focuses on what is meaningful to the individual [53], to create a care scenario that is sensitive to the individual’s needs was described:

“A very good approach, because when we were talking the problems, …It gets us to narrow things down to talk about what’s important.”

(nurse) [55, p. 884].

Taking a person-centred approach also helps care providers to ‘see’ individuals instead of homogenizing older adults and treating them collectively as a “grey mass of pensioners” [65]. This was also evident in relation to the use of screening facilitating the change of frail people from invisible to visible and in need of support, in the perceptions of the clinicians: *“They are visible now*. *Before the programme we were not confronted with these patients*. *However*, *I am more aware of this type of patient now*.*”* (GP) [36, p. 2266].

#### Power and choice

Eleven papers contributed to this theme [40,41,43,44,48,49,53,54,65,67,70]. Challenges faced by many older adults and care providers in the included studies were related to the diminished power they experienced in their relationships with services and the community. Frail older people reported that they felt de-personalized in several ways, and automatically judged as incompetent:

“…when you are treated is if you’ve become a child again. It is hurting when they think that older people can’t hear well and can’t fill in forms. [..] Older persons are treated as children. You become incapacitated because you are older. That is unacceptable”.

(frail older adult) [65, p. 7].

This stereotyped way of depicting the frail older person could result in neglecting personal resources and hampering actions or strategies older persons may be willing to take themselves:

“[…] then I asked my contact person […] I said you have to make the call [..] otherwise they will not believe me. They believe […] there are many people who will take us for persons with dementia just because we live here (resident of a former care home for the elderly who needs to contact a service because the doorbell has a weak signal)”

(frail older person) [43, p. 95].

The quality of the relationship between older adults and care providers is significant in maintaining older adults’ sense of control over their decision-making, and, essentially, the sense of their personal value [54]. They described the encounter with health and social care as being largely concerned with the struggle for such control, and how this had implications for the frail older adults’ satisfaction with care, but also for their self-esteem, with frustration being expressed when choices were made according to preferences and standpoints determined by other people or by the system of care.

“[…] unfortunately we cannot influence that, because they decide in their office [the home care service decides] under what conditions we are going out for a walk, and when we should, when they should shop.”

(frail older person) [43, p. 94]

Not being involved in decisions was perceived as even more threatening for the person’s integrity, like not being respected:

“But I have a feeling that they are not listening […] and they think that we are old and have no right to make decisions […] really […] […] but maybe it doesn’t have to do with the right to make decisions, this experience I have of being older. You do not get respect!”

(frail older person) [43, p. 94]

Some studies addressed the point of view of professionals in this potentially disempowering encounter with frail older people and found that staff's attitudes can result in them giving up taking even the smallest decisions:

“Yes, a lot of them have said, ‘Well, what do you want me to do now?’ or ‘You know better’, ‘If that’s what you want dear.’ But to be honest I was quite happy to do what I want to do for them, but lately I’ve been trying to encourage them and one lady especially, she was saying, ‘Well, what am I going to do now?’ and I said, ‘Well what would you like to do?’ and she said, ‘Well, I’ve finished using the commode’ and then I said, ‘So, what do you want to do now?’ and she said ‘Well, I don’t know, you have to tell me because I don’t know what to do, you always tell me what to do, I’m not in the position to tell you what I have to do.’ And I think I was shocked with that conversation entirely.”

(nurse) [70, p. 146]

These findings illustrate the central importance of the issue of power. Frailty was seen as a condition challenging the possibility to go on living according to personal preferences and the person’s sense of identity, in spite of frailty. The point was not being overwhelmed by difficulties, but also finding a way to constant growth and satisfaction, and this is possible when people can cope with the dramatic changes frailty causes.

## Discussion

**Fig 2:**
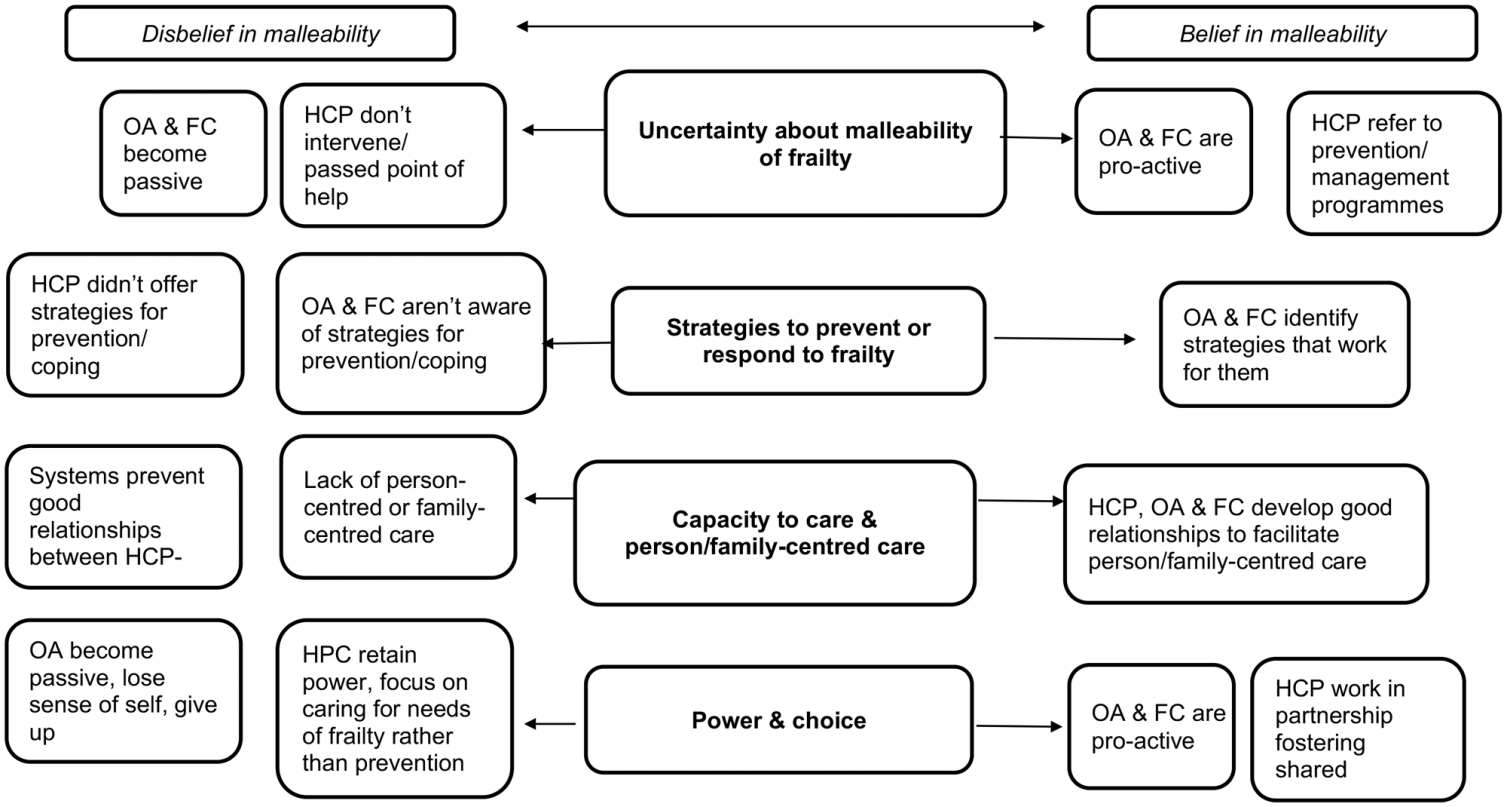
Model of frailty from different stakeholders’ perspectives (HCP: health & care professionals; OA: older adults; FC: family carers).

This meta-synthesis included the points of view of different stakeholders: frail and non-frail older people, caregivers, and health and social care professionals. We generated four synthetic themes (third-order constructs) across them, depicted in our tentative model of frailty (Fig 2). *Uncertainty about the malleability of frailty* showed that becoming frail was not universally perceived as preventable and amenable to change across all stakeholders or at all stages. Reflecting on the synthesized findings, this theme represented a pivotal belief which then impacted on the nature of the remaining themes and determined different paths in the response to frailty. Thus, a belief or disbelief in the malleability of frailty took up a central position in the model. This *uncertainty about the malleability of frailty* represents an awareness of being in transition, drawing on the Third and Fourth Age dialectic, which finds older adults inhabiting a liminal space between dependence and independence, activity and passivity [75].

The theme of *Capacity to care* took a central position too. The potential vulnerability within this uncertainty demands flexible services and a belief in the malleability of frailty on behalf of health and social care professionals to deal with it. Nicholson et al. (2012) [9] say that "welfare provision separates people into either living or dying in order to determine care needs", using a binary classification which forces the significance of older people’s experience of accumulated loss into an all-or-nothing scheme which precludes the development of individual routes toward coping and adjustment. This theme also revealed problems in current health and social care systems but also ideals about what different stakeholders believed constituted good care.

Communication, information, quality of relationships and, importantly, the perceived lack of these specific skills among health and social care providers were critical issues according to all stakeholders. Family carers wanted to be involved in the process and health and social care providers needed their involvement for services to be accessed. The role of the family, the central resource it represents for the care and the quality of life of frail older people, was therefore recognised in the evidence base. Although initiatives exist [76], more work is required to meet the needs of families. For older adults and family caregivers to become pro-active, the system of care should foster belief in the malleability of frailty through the appreciation and use of individual and family coping strategies. In fact, under the area of malleable frailty there was the scope for identification of useful strategies which were largely offered by older adults and family caregivers rather than health and social care professionals. Older adults identified a number of strategies which worked for them and were appropriate to their level of ability, multiple conditions they may have, and their surroundings in terms of their home and local environment. The strategies mentioned by the frail older people were characterised by common utilisation of a “loss based selection” approach being used [77]. Baltes & Baltes’ definition of successful ageing is useful here: “the resilience of people who succeed in achieving a positive balance between gains and losses during aging … not just coping with decline, but they continue to actively develop themselves on numerous fronts.”

People’s response to losses, such as everyday functional abilities or mobility is what distinguished people who managed to maintain well-being, from those who did not. The Selective Optimisation and Compensation (SOC) model [78] suggests an approach for explaining the way individuals maximised their potential gains, minimised potential losses by adaptive selection of goals and optimised their route towards selected goals. Within the SOC model is the concept of acceptance, which was powerful in older adults’ accounts; older adults selected as targets the goals they perceived and accepted as changeable and focused on those in order to exert some control over their management of frailty.

The theme of *Power and choice* was connected to the previous ones in the way that the provision of person- and family-centred care places the person and family unit and their needs at the centre in order to generate care which respects personhood, fosters control on the condition, facilitate successful relationships between stakeholders, and enables older adults and their caregivers to engage in shared decision-making. This is in line with what we know about concordance [79]. Respect, equipoise, and accessible information are required for health and social care professionals, older adults, and their families to embark on a process of shared decision-making to develop a personalised care plan [80].

The third-order constructs generated in this synthesis point toward the need for a lifeworld-led care perspective [81]. Lifeworld-led care involves a humanising practice of care with participation of, in this case, older adults and their families. The foundation of lifeworld-led care is to take account of how everyday experiences, individuals’ values and the meanings they attach to their experiences, like becoming frail and receiving care, together with contextual factors such as the home and local environment, can be used to inform care. Qualitative research can play a crucial role in this, and this review has demonstrated several elements which could eventually contribute to it. For instance, interventions need to be explicitly at the family level because so many interventions depend on that informal support. Care should be holistic, grounded in the real context of older people and families, and built upon solid relationships between care providers and older adults/caregivers, and abilities to work together for common goals.

### Strengths and limitations

In order to ensure methodological quality of our review, the searching and screening process was based on pre-specified criteria, and the assessment of methodological quality of each paper was made by pairs of independent reviewers. Disagreements between reviewers about inclusion were resolved through discussion. The use of quality appraisal tools did not entail any paper exclusion. There are doubts about the meaning of quality appraisal in meta-synthesis, since it is based on methodological and formal aspects and can neglect the more insightful dimension of the paper [82]. This can be a serious problem, anyway we wanted to have an idea of how the included papers performed in face of some criteria.

The synthesis process was shared between team members and decisions about themes were discussed in order to demonstrate transparency between original studies and our synthesis of them.

However, we could not manage all difficulties. The study was restricted to literature existing in three electronic databases. Also, we chose to include only papers in the languages known by the group members, thus excluding papers that could have provided important insights. Connected to this, we acknowledge that the cultural representation of stakeholders in the reviewed studies was limited.

Publication bias could affect our results. It was shown that publication bias in qualitative research can cause a large number of studies presented in conference settings or other contexts remain in a drawer [83]. Looking for these reports and other grey literature could help address this bias. We limited our search to published papers given the high number retrieved. Although not including grey literature could be viewed as a limitation of this work, its inclusion would have made it unmanageable.

The reviewed studies were based on different epistemological models and different methodological designs, with their internal validity dependent on the framing adopted for the research. This necessarily raises the question of the possibility to gather them in a comprehensive synthesis. However, this difficulty is inherent to synthesis of qualitative (as well as quantitative) studies, which are all individually different [84]. To this regard, we agree with Finfgeld (2003) [85] who has argued that the combination of findings from multiple approaches can enhance the ‘truth value’ of the synthesis. Meta-ethnography is a widespread methodology employed to synthesise different and numerous studies, illustrating its suitability to a review data-set like this one [23,25].

Other points are related more to the limits of the included papers. We cannot rule out the possibility of selection biases in the included studies affecting the results of our review. The included studies also presented some methodological weaknesses. Few authors provided information about strategies used to control bias of subjectivity and discussion about saturation of data was frequently omitted. The impact of taking part in the research on the participants was only rarely considered, although some acknowledged that the process itself had useful outcomes [56].

Moreover, the definition of frailty used to select participants was not universally given, and among those who did there was huge variation—from use of a full geriatric assessment, use of a frailty phenotype, to selecting people who received support from a local service or who had been admitted to hospital on more than one occasion in a set period. Nevertheless, this should not have an impact on the findings given that our aim was to examine understandings, attitudes and values towards frailty and interventions and care for frailty.

## Conclusion

If we want frailty to be approached as a malleable and preventable condition, a bottom-up approach is needed. Not only should the real needs of frail older people and carers be listened to, but also the tools themselves through which frailty can be managed should come from their own context and resources. This lifeworld-led care paradigm means involvement of the person and family in identification of needs and intervention, enhancement of useful coping strategies, and addressing of the negative ones. The absence of qualitative studies involving psychologists suggests that future research is needed to address their point of view about their own role in support of prevention and intervention. Although several papers addressed the importance of the relationship in the process of care and in the person-centred approach, health and social professionals still need to understand the meaning of building relationship in the context of care. This should be addressed in qualitative research to be better approached in health and education settings.

Qualitative research can prompt awareness in professionals, about their behaviour and how the system influences attitudes and choices. None of the papers included in this review had addressed policy makers’ views of pre-frailty and frailty. An important contribution to the discussion about implementation of real and systematic actions for early detection of frailty and prevention of its progression is missing and represents a need for further research.

## Supporting information

S1 Text Search strategy. (DOCX)

S2 Text CASP-qualitative-research-checklist. (PDF)

S1 Table Table of characteristics of included studies. (DOCX)

S2 Table PRISMA 2009 checklist. (DOC)

S3 Table Table of summary of findings and conclusions from included studies. (DOCX)
