# Correction: Stakeholders' views and experiences of care and interventions for addressing frailty and pre-frailty: A meta-synthesis of qualitative evidence

**DOI:** 10.1371/journal.pone.0191763

**Published:** 2018-01-19

**Authors:** Barbara D’Avanzo, Rachel Shaw, Silvia Riva, Joao Apostolo, Elzbieta Bobrowicz-Campos, Donata Kurpas, Maria Bujnowska-Fedak, Carol Holland

The seventh author's name appears incorrectly. The correct name is: Maria Bujnowska-Fedak.
