# Supplementary material for: Stakeholders’ views and experiences of care and interventions for addressing frailty and pre-frailty: A meta-synthesis of qualitative evidence
Source: PLoS One. 2017 Jul 19;12(7):e0180127. doi: 10.1371/journal.pone.0180127 (PMC5516973; doi:10.1371/journal.pone.0180127)
Supplement: S1 Text — (DOCX) [file pone.0180127.s001.docx]

|  |  | **S1 Text. Search strategy**  "Frail Elderly"[Mesh] OR older adult*[Title/Abstract] OR old*[Title/Abstract] OR elder*[Title/Abstract]  AND  frail*[Title/Abstract]  AND |  |
| --- | --- | --- | --- |

"Qualitative Research"[Mesh] OR "Nursing Methodology Research"[Mesh] OR "Focus Groups"[Mesh] OR "Interviews as Topic"[Mesh] OR patients perspective*[Title] OR patient experience*[Title]) OR "lived experience"[Title] OR attitude[Title] OR "point of view"[Title] OR "points of view"[Title] OR patient public involvement*[Title/Abstract] OR "qualitative research"[Title/Abstract]OR interview*[Title/Abstract] OR "focus group"[Title/Abstract] OR "focus groups"[Title/Abstract] OR perception*[Title] OR viewpoint*[Title] OR need*[Title] OR expectation*[Title] OR "mixed method"[Title/Abstract] OR "mixed methods"[Title/Abstract]

We searched Pubmed (June 2015) and this search strategy was then adapted to CINHAL and Web of Science.
