# Supplementary material for: Stakeholders’ views and experiences of care and interventions for addressing frailty and pre-frailty: A meta-synthesis of qualitative evidence
Source: PLoS One. 2017 Jul 19;12(7):e0180127. doi: 10.1371/journal.pone.0180127 (PMC5516973; doi:10.1371/journal.pone.0180127)
Supplement: S1 Table — (DOCX) [file pone.0180127.s003.docx]

## S1 Table. Table of the characteristics of included studies

| **Reference** | **Research question/objectives** | **Location** | **Sample** | **Methods** | **Quality rating*** |
| --- | --- | --- | --- | --- | --- |
| Ayalon L, et al. Social workers' perspectives on care arrangements between vulnerable elders and foreign home care workers: lessons from Israeli/Filipino caregiving arrangements. Home Health Care Serv Q 2008;27(2): 121-142. | Evaluates the advantages and challenges associated with home arrangement of care from the perspective of social workers (Filipinos working as home care providers in Israeli homes) | Israel | Social workers | Focus group | SAT |
| Baillie L, et al. Care transitions for frail, older people from acute hospital wards within an integrated healthcare system in England: a qualitative case study. Int J Integr Care 2014;14: e009. | To investigate the care transitions of frail older people from acute hospital wards to community healthcare or community hospital rehabilitation wards, within the context of a healthcare system that had vertically integrated acute hospital and community healthcare services. | UK | Physiotherapists, social workers, nurses, occupational therapists | Interviews and focus group | SAT |
| Bindels J, et al. Care for community-dwelling frail older people: a practice nurse perspective. J Clin Nurs 2014;23(15-16): 2313-2322. | To evaluate care programmes for community-dwelling frail older people from a practice nurse perspective and gain a deeper understanding of their role within the programmes | Netherlands | Health professionals | Interviews and observation | SAT |
| Bindels J, et al. Losing connections and receiving support to reconnect: experiences of frail older people within care programmes implemented in primary care settings. Int J Older People Nurs 2015;10(3): 179-189 | To evaluate whether care provided in the care programmes matched the needs of older people | Netherlands | Frail older people | Interviews and focus group | SAT |
| Blanton PW. Family Caregiving to Frail Elders: Experiences of Young Adult Grandchildren as Auxiliary Caregivers. Journal of Intergenerational Relationships 2013;11(1): 18-31. | To describe the essence of the lived experiences of young adult grandchildren as auxiliary caregivers in the process of family caregiving to frail grandparents. | US | Caregivers (grandchildren) | Interviews with families | SAT |
| Bleijenberg N, et al. Exploring the expectations, needs and experiences of general practitioners and nurses towards a proactive and structured care programme for frail older patients: a mixed-methods study. J Adv Nurs 2013;69(10): 2262-2273. | To report the expectations and experiences of GPs and nurses regarding the U-CARE programme, to gain a better understanding of the barriers and facilitators in providing proactive, structured care to frail older people and to determine whether implementation is feasible | Netherlands | Health professionals (GPs and practice nurses, PNs) | Focus group | SAT |
| Claassens L, et al. Perceived control in health care: a conceptual model based on experiences of frail older adults. J Aging Stud 2014;31: 159-170. | To investigate the concept of health care-related perceived control from the viewpoint of frail older adults. | Netherlands | Frail older people | Interviews and focus group | SAT |
| Denson LA, et al. Discharge-planning for long-term care needs: the values and priorities of older people, their younger relatives and health professionals. Scand J Caring Sci 2013;27(1): 3-12. | To compare the opinions and values of frail elders living at home, younger relatives and health professionals experienced in discharge-planning, prospectively: before, not after, a long term care decision. | Australia | Frail older people,  relatives and health professionals | Interviews | KP |
| Dick K and Frazier SC. An exploration of nurse practitioner care to homebound frail elders. J Am Acad Nurse Pract 2006;18(7):325-334. | To identify and classify care activities of nurse practitioners providing primary care for frail homebound elders and to describe NPs' perceptions of the outcomes of their care activities | US | Nurses | Interviews and focus group | SAT |
| Donlan WT. The meaning of community-based care for frail Mexican American elders. International Social Work 2011;54(3): 388-403. | The rapid increase and the higher rates of disability and the underutilization of services by Mexican older people suggest the need to illuminate the complex social and cultural context of the experience of frail Mexican-origin frail elders receiving assistance. | US | Frail older people | Interviews and observation | SAT |
| Ebrahimi Z, et al. Frail elders' experiences with and perceptions of health. Qual Health Res 2012;22(11): 1513-1523. | To explore frail elders' experiences with and perceptions of the phenomenon of health so as to develop a deeper understanding of living with diseases and disorders in old age. | Sweden | Frail older people | Interviews | KP |
| Ebrahimi Z, et al. Health despite frailty: exploring influences on frail older adults' experiences of health. Geriatr Nurs 2013;34(4): 289-294. | To explore and identify influences on frail older adults' experience of health. Adults taken from a broader sample from a quantitative study on health. | Sweden | Frail older people | Interviews | KP |
| Ekelund C, et al. Self-determination among frail older persons -- a desirable goal older persons' conceptions of self-determination. Quality in Ageing & Older Adults 2014;15(2): 90-101. | To explore older persons' different conceptions of self-determination. | Sweden | Frail older people | Interviews | SAT |
| Ekwall A, et al. Compensating, controlling, resigning and accepting-older person's perception of physical decline. Curr Aging Sci 2012;5(1): 13-18. | To know about how frail older people experience their physical decline and how they adapt to their bodily changes so that the health system can design preventive interventions targeting this group early on in the disability process. The aim of this study was to explore how older people perceive their physical decline. | Sweden | Frail older people | Interviews | SAT |
| Faes MC, et al. Qualitative study on the impact of falling in frail older persons and family caregivers: foundations for an intervention to prevent falls. Aging Ment Health 2010;14(7): 834-842. | To explore the impact of falling for frail community-dwelling older persons with and without cognitive impairments who have experienced a recent fall and their primary family caregivers | Netherlands | Frail older people and caregivers | Interviews | SAT |
| Fjelltun, AM, et al. Nurses' and carers' appraisals of workload in care of frail elderly awaiting nursing home placement. Scand J Caring Sci 2009;23(1): 57-66. | To describe carers' and nurses' appraisals of workload in care of frail elderly awaiting nursing home placement. | Norway | Nurses and caregivers | Interviews | SAT |
| Fjelltun AS, et al. Carers' experiences with overnight respite care. A qualitative study. Nordic Journal of Nursing Research & Clinical Studies / Vård i Norden 2009;29(3): 23-27. | To explore experiences with overnight respite care of Norwegian carers who provided care to frail elderly awaiting nursing home placement. | Norway | Caregivers | Interviews | SAT |
| Grenier A and Hanley J.  Older women and 'frailty' - Aged, gendered and embodied resistance.  Current Sociology 2007;55: 211-28. | This article draws on complex forms of resistance of older women to the concept of frailty and role of frail person. | Canada | Frail older people | Interviews | SAT |
| Gustafsson S, et al. Swedish Health Care Professionals' View of Frailty in Older Persons. Journal of Applied Gerontology 2012;31(5): 622-640. | To elucidate health care professionals' view of frailty in older persons. | Sweden | Health professionals | Focus group | SAT |
| Hjaltadottir I and Gustafsdottir M. Quality of life in nursing homes: perception of physically frail elderly residents. Scand J Caring Sci 2007;21(1): 48-55. | To disclose the characteristics of quality of life as perceived by physically frail but lucid elderly people living in nursing homes to increase the understanding of the phenomenon of quality of life in this setting. | Iceland | Frail older people | Interviews | SAT |
| Horder HM, et al. Self-respect through ability to keep fear of frailty at a distance: successful ageing from the perspective of community-dwelling older people. Int J Qual Stud Health Well-being 2013;8: 20194. | To explore successful ageing from the perspective of community-dwelling older people (24 persons aged 77-90 years). | Sweden | Frail older people | Interviews | SAT |
| Kita M and K Ito. The caregiving process of the family unit caring for a frail older family member at home: a grounded theory study. Int J Older People Nurs 2013;8(2): 149-158. | To explore the caregiving process of family units caring for a frail older family member at home. | Japan | Caregivers | Interviews with families | SAT |
| Koenig TL. From the woman's viewpoint: Ethical dilemmas confronted by women as informal caregivers of frail elders.  Families in Society-the Journal of Contemporary Human Services 2004;85: 236-42. | To explore women's ethical decision-making in caring for a frail elder. | US | Caregivers | Interviews | SAT |
| Kristensson J, et al. Frail older adult's experiences of receiving health care and social services. J Gerontol Nurs 2010;36(10): 20-28; quiz 30-21. | To explore frail older adults' overall experience of receiving health care and/or social services. | Sweden | Frail older people | Interviews | KP |
| Levesque L, et al. A partnership approach to service needs assessment with family caregivers of an aging relative living at home: a qualitative analysis of the experiences of caregivers and practitioners. Int J Nurs Stud 2010;47: 876-887. | To explore the experiences of caregivers and practitioners who took part in a field test of the Family Caregivers Support Agreement tool, designed to facilitate partnerships between caregivers and practitioners so that needs assessment and subsequent support services are negotiated and agreed so as to meet caregiver expectations. | Canada | Caregivers, nurses, social workers | Focus group | SAT |
| Lindhardt T, et al. Nurses' experience of collaboration with relatives of frail elderly patients in acute hospital wards: a qualitative study.  Int J Nurs Stud 2008;45: 668-681. | To illuminate nurses' experience of collaboration with relatives of frail elderly patients in acute hospital wards, and of the barriers and promoters for collaboration. | Denmark | Nurses | Interviews | KP |
| McGeorge SJ. Unravelling the differences between complexity and frailty in old age: findings from a constructivist grounded theory study. J Psychiatr Ment Health Nurs 2011;18(1): 67-73. | To explore findings from a study into how mental health nurses who work with older people construct and operationalize the concept of 'age-related complexity'. This paper addresses the relationship between frailty and complexity, which was identified as a theme within the category 'dynamic complexity'. | UK | Nurses | Interviews | KP |
| Modig S, et al. Frail elderly patients' experiences of information on medication. A qualitative study. BMC Geriatr 2012;12: 46. | To explore frail elderly patients' experiences of receiving information about their medications and their views on how the information should best be given. | Sweden | Frail older people | Interviews | SAT |
| Nicholson C, et al. Living on the margin: understanding the experience of living and dying with frailty in old age. Soc Sci Med 2012;75(8): 1426-1432. | Understanding of the experience and challenges of living and dying with frailty in older age. | UK | Frail older people | Interviews | KP |
| Nicholson C, et al. The experience of living at home with frailty in old age: a psychosocial qualitative study. Int J Nurs Stud 2013;50(9): 1172-1179. | To understand the experience over time of home-dwelling older people deemed frail, in order to enhance the evidence base for person-centred approaches to frail elder care. | UK | Frail older people | Interviews and observation | KP |
| Puts MT, et al. The meaning of frailty according to Dutch older frail and non-frail persons. Journal of Aging Studies 2009;23(4): 258-266. | To explore the meaning of quality of life to older frail and non-frail persons living in the community. | Netherlands | Frail older people | Interviews | KP |
| Puts MT, et al. What does quality of life mean to older frail and non-frail community-dwelling adults in the Netherlands? Qual Life Res 2007;16(2): 263-277. | To describe the meaning of quality of life from the perspective of frail and non-frail older community dwelling persons. | Netherlands | Frail and non-frail older people | Interviews | SAT |
| Robben S, et al. Preferences for receiving information among frail older adults and their informal caregivers: a qualitative study. Fam Pract 2012;29(6): 742-747. | To explore the experiences of frail older people and informal caregivers with receiving information from health care professionals as well as their preferences for receiving information. | Netherlands | Frail older people and caregivers | Interviews | KP |
| Roland KP, et al. Exploring Frailty: Community Physical and Occupational Therapists' Perspectives. Physical & Occupational Therapy in Geriatrics 2011;29(4): 270-286. | To explore therapists' perspectives on frailty, and develop a definition of how they view and manage frailty in their practice. | Canada | Occupational therapists | Interviews and focus group | SAT |
| Rush KL, et al. Older adults' perceptions of weakness and ageing. Int J Older People Nurs 2013;8(1): 1-9. | To understand the meaning of weakness for older adults' and their perceptions of its association with ageing. | Canada | Frail older people | Interviews | KP |
| Sarvimaki A and Stenbock-Hult B. The meaning of vulnerability to older persons. Nurs Ethics 2014. | To illuminate the meaning of vulnerability. | Finland | Frail older people | Interviews | KP |
| Skymne C, et al. Getting used to assistive devices: ambivalent experiences by frail elderly persons. Scand J Occup Ther 2012;19(2): 194-203. | To learn how frail elderly people experienced becoming assistive device users and how assistive devices affected their independence in daily activities. | Sweden | Frail older people | Focus group | KP |
| Stockwell-Smith G, et al. Why carers of frail older people are not using available respite services: an Australian study. J Clin Nurs 2010;19(13-14): 2057-2064. | To explore the limiting and motivating factors that influence carers' use of respite services and the ability of currently available respite services to meet the needs of carers of frail older people | Australia | Caregivers | Focus group | SAT |
| Teixeira IN. [The perception of health professionals of two definitions of frailty in elderly people]. Cien Saude Colet 2008;13(4): 1181-1188. | To understand the perception of health professionals of two definitions of frailty in elderly people | Brazil | Health professionals | Interviews | SAT |
| Themessl-Huber M, et al. Frail older people's experiences and use of health and social care services. J Nurs Manag 2007;15(2): 222-229. | To highlight older people's experiences and expectations of services and the consequences for service provision, service development and research. | UK | Frail older people | Interviews | KP |
| Tutton EM. Patient participation on a ward for frail older people. J Adv Nurs 2005;50(2): 143-152. | To explore the meaning of participation for older people in hospital and their health care workers and ways in which staff can enhance patient participation in their care | UK | Health professionals and frail older people | Interviews and focus group | SAT |
| van Kempen JA, et al. Home visits for frail older people: a qualitative study on the needs and preferences of frail older people and their informal caregivers. Br J Gen Pract 2012;62(601): e554-560. | To explore the views and needs of community-dwelling frail older people concerning home visits | Netherlands | Frail older people | Interviews | SAT |
| Walker R, et al. How older people cope with frailty within the context of transition care in Australia: implications for improving service delivery. Health Soc Care Community 2015;23(2): 216-224. | Examines how older people cope with frailty within the context of a dedicated transition care programme and discusses implications for improving service delivery | Australia | Frail older people | Interviews | SAT |
| Wallin M, et al. Physiotherapists' accounts of their clients in geriatric inpatient rehabilitation. Scand J Caring Sci 2008;22(4): 543-550. | To explore and describe the consequences of an acute hip fracture as experienced by home-dwelling elderly people shortly after discharge from hospital. | Sweden | Physiotherapists | Interviews | KP |
| Zidén L, et al. A life-breaking event: early experiences of the consequences of a hip fracture for elderly people. Clin Rehabil 2008;22(9): 801-811. | To explore and describe the consequences of an acute hip fracture as experienced by home-dwelling elderly people shortly after discharge from hospital. | Sweden | Frail older people | Interviews | KP |

*KP: Key paper – meets all quality criteria and clearly fits with review question; SAT: satisfactory – meets most quality criteria and fits well to review question; UNS: unsure – mixed responses to quality criteria and lack of clarity regarding relevance to review question; P: poor – does not meet quality criteria
