# Supplementary material for: Stakeholders’ views and experiences of care and interventions for addressing frailty and pre-frailty: A meta-synthesis of qualitative evidence
Source: PLoS One. 2017 Jul 19;12(7):e0180127. doi: 10.1371/journal.pone.0180127 (PMC5516973; doi:10.1371/journal.pone.0180127)
Supplement: S3 Table — (DOCX) [file pone.0180127.s005.docx]

**S3 Table. Table of summary of findings and conclusions from included studies**

| **Reference** | **Aim, participants and location** | **Findings** | **Authors' implications and conclusions** |
| --- | --- | --- | --- |
| Ayalon L, et al. Social workers' perspectives on care arrangements between vulnerable elders and foreign home care workers: lessons from Israeli/Filipino caregiving arrangements. Home Health Care Serv Q 2008;27(2): 121-142. | Evaluates the advantages and challenges associated with home arrangement of care from the perspective of social workers (Filipinos working as home care providers in Israeli homes)  Social workers  Israel | Motivation for the arrangement are money for the Filipinos and absence of alternative for those who want to stay at home as long as possible for the older Israeli; there is fear of foreign care entering the home, but still the Israeli want it; some families would like the home carer become part of the family. On a whole, the home care arrangement brings loneliness to both parts. Filipinos work just for money and "have no affects". Both parts feel abused, physically and economically. There are also cultural and culinary differences. | It is important to involve additional supervising bodies that aspire to protect both the older adults and the foreign home care workers and favouring caregiving arrangement.  Social workers can have an important role in preparation and matching of family and home carer, supervision, emotional support to both parts. |
| Baillie L, et al. Care transitions for frail, older people from acute hospital wards within an integrated healthcare system in England: a qualitative case study. Int J Integr Care 2014;14: e009. | To investigate the care transitions of frail older people from acute hospital wards to community healthcare or community hospital rehabilitation wards, within the context of a healthcare system that had vertically integrated acute hospital and community healthcare services.  Physiotherapists, social workers, nurses, occupational therapists  UK | Interprofessional communication and relationships and patient and family involvement in care transitions. More in particular: boundaries at staff level remained; community staff often perceived the acute hospital as being separate from their services and they believed that acute ward staff did not understand community provision and roles. The study revealed delays in care transitions from acute wards but also frequent moves of older people between acute wards. Lack of capacity in community health and social care provision was perceived as a barrier to transitions and capacity or resource issues are acknowledged. At ward level, staff participants discussed how they involved patients and families in planning for transitions but findings also revealed some lack of communication and involvement. Decisions about transitions were often made quickly to increase bed availability in acute wards, but a quick discharge may affect the quality of service that staff can offer and patients can be left feeling worried and distrusting. | Most staff working directly with patients were based either in settings which remained separated, and there were few opportunities for them to build relationships and to develop understanding of service provision and roles in other parts of the system. The removal of organizational boundaries does not necessarily reduce boundaries between staff at interpersonal level and enable staff in different settings to work together effectively. Opportunities for staff to rotate between settings, and the establishment of forums for staff to build relationships and develop understanding of others’ roles and of other settings and their services, could assist staff to work in a more integrated way. There is need to develop pathways for frail older people that prevent repeated moves around acute hospital wards, and the importance of effective communication with patients and their families about transitions.  Nurses could be in a good position to support and empower patients as they transition between hospital and long-term settings but  ward staff may lack control in relation to care transitions and disempowered staff may not feel well placed to empower patients. |
| Bindels J, et al. Care for community-dwelling frail older people: a practice nurse perspective. J Clin Nurs 2014;23(15-16): 2313-2322. | To evaluate care programmes for community-dwelling frail older people from a practice nurse perspective and gain a deeper understanding of their role within the programmes  Health professionals  Netherlands | For PNs (practice nurses), there are 4 main themes  in activating care programmes for community-dwelling frail older people that need to be considered:  ‘building a trusting relationship’ and ‘making connections’ with patients, family members and other professionals which are key elements in detecting problems and providing care for frail older people. The other themes are ‘targeting the wrong audience?’ and ‘providing good care’ which represent  the considerations of PNs have with regard to the care they provide and the selection procedures. PNs perceive that many older people who are enrolled in the programme appear not to be frail.  Practice nurses feel that a majority of people in the programme are not frail or are already receiving appropriate care. Optimal screening instrument (able to detect particularly the frailest older people who do not respond to the screening questionnaire due to physical or cognitive impairments or due to care avoidance behaviour) has not yet been found. | More research is needed to develop interventions and strategies that can support PNs in targeting loneliness in older people. |
| Bindels J, et al. Losing connections and receiving support to reconnect: experiences of frail older people within care programmes implemented in primary care settings. Int J Older People Nurs 2015;10(3): 179-189 | To evaluate whether care provided in the care programmes matched the needs of older people  Frail older people  Netherlands | Two categories emerged from the data: ‘Losing connections’ and ‘Receiving support to reconnect.’ ‘Losing connections’ reflects the needs of older people and ‘Receiving support to reconnect’ reflects their experience and the appreciated aspects of the provided care. A relationship of trust with the practice nurse (PN) appeared to be an important aspect of care, as it fostered the sharing of feelings and issues other than physical or medical problems that could not be shared with the general practitioner. Although the PNs play a very medical role, they are experienced as connectors, who help to restore feelings of connectedness and older peoples’ access to resources in the community.  Waiting to die; feeling that they were not useful to others. | A proactive approach to detect health problems in community-dwelling older people can lead to an increased detection of problems in the social domain, including feelings of losing connections. Care programmes including home visits by a practice nurse for frail older people in the community can help to restore feelings of connectedness and older peoples’ access to resources in the community. A trusting relationship between a practice nurse and a frail older person is crucial for the receptiveness of care and functions as the basis of care for frail older people. Nurses can fulfil an essential role in community-based |
| Blanton PW. Family Caregiving to Frail Elders: Experiences of Young Adult Grandchildren as Auxiliary Caregivers. Journal of Intergenerational Relationships 2013;11(1): 18-31. | To describe the essence of the lived experiences of young adult grandchildren as auxiliary caregivers in the process of family caregiving to frail grandparents.  Caregivers (grandchildren)  US | Intergenerational relationships were described as being either relatively high on solidarity and low on conflict or relatively low on solidarity and high on conflict. There was continuity in these differing dynamics of relating, but the process of caregiving seemed to highlight in more intense ways the preexisting connections and difficulties in intergenerational relationships in families.  Grandchildren contributed to family caregiving primarily providing emotional support, although a few also in activities of daily living. Grandchildren were aware that their demands for physical care were much less than those of the primary caregiver. A common theme was a focus on trying to bolster the spirits of their grandparents, creating pleasant experiences for them, and offering them emotional support. Various ways of interacting with grandparents were described as strategies for achieving these hoped for outcomes: “I love for her to laugh and so that is basically what I try to do. We go walk and I sing with her.” Most grandchildren felt rewarded by the pleasurable moments they helped to create for and share with their grandparents. There was also an awareness of the greater emotional burden of experiencing losing a parent for the primary caregiver. “For my father, it is his father that is dying and I think it is more emotionally hard for him.” Several of the participants spoke of feeling caught in the middle or pulled, in some way or another, in the family caregiving situations in which they were involved. Their awareness of these tensions created for them, at times, feelings of frustration. Those grandchildren who described higher levels of conflict coupled with low solidarity in intergenerational relationships tended to emphasize the more burdensome and stressful aspects of the process of family caregiving. |  |
| Bleijenberg N, et al. Exploring the expectations, needs and experiences of general practitioners and nurses towards a proactive and structured care programme for frail older patients: a mixed-methods study. J Adv Nurs 2013;69(10): 2262-2273. | To report the expectations and experiences of GPs and PNs regarding the U-CARE programme, to gain a better understanding of the barriers and facilitators in providing proactive, structured care to frail older people and to determine whether implementation is feasible  Health professionals (GPs and practice nurses, PNs)  Netherlands | The GPs reported difficulties in providing coordinated care to frail older patients due to a lack of time. Furthermore, GPs indicated that another limitation was a lack of a well-educated PN and no financial compensation to provide this care. The PNs of this study indicated positive experiences.as  nurses.  Half of the PNs questioned whether the programme was beneficial for all patients. They were uncertain about the programme’s effectiveness for patients who were not motivated, were less open to change or had little knowledge of relevant problems. | The results of this study show that implementation of proactive care programmes in clinical practice is complex by nature. To improve implementation, they have defined five preconditions that must be fulfilled based on our results. First, the providers of the new intervention must be well educated and trained. Second, to enhance the quality and feasibility of a new care programme in clinical practice, the providers of the intervention must be involved during the development phase of the intervention. Third, financial compensation for the proactive preventive care is required. Fourth, good collaboration between GPs and PNs is needed to improve care for older people. Fifth, it is important for researchers and innovators to acknowledge that it takes time for new care programmes or models to be adapted by providers and to ensure that the intervention works in the most efficient and effective way. |
| Claassens L, et al. Perceived control in health care: a conceptual model based on experiences of frail older adults. J Aging Stud 2014;31: 159-170. | To investigate the concept of health care-related perceived control from the viewpoint of frail older adults.  Frail older people  Netherlands | Perceived control reflects the feeling or the belief that health care is under control, arising from multiple constituting factors: self-confidence in organising professional and/or informal care, self-confidence in health management in the home setting (self-management: self-care; health-related behaviour, also preventive; level of discipline & order; predictability of & familiarity with health problems or disease symptoms; coping strategies with regard to irreversible and/or adverse health & health care outcomes: acceptance, positivism, perseverance, forgiveness, participation), perceived support from people in the social network (presence and availability of people in the social network; receiving their support at different domains of care; responsiveness of these informal helpers to one’s wishes and need for autonomy), health care professionals and organisations (goal-oriented and commitment in the health problems, accessibility of organisations with clear contact points, relationship of trust, coordination, efficiency, responsiveness to wishes/autonomy client, stability of rules/policy, and health care infrastructure and services).  The findings indicated that the level of perceived control was dependent on the factors that constitute it: when all factors were favourable (sufficient support possibilities as well as confidence in own abilities), perceived control was high. Whenever some factors were less favourable, respondents appealed to their strengths or beneficial circumstances, i.e. to those factors that were developed more strongly, in order to compensate for the weaker ones. | The meaning of control to our respondents appeared to be relative to their functional abilities; even the respondents with poor functional abilities stated that they had some degree of personal control (e.g. to ‘think along’). Therefore, perceived control can refer to full control in both thinking and acting, but also to less active forms of control, where only cognitive processes play a role. Although in general independence was highly valued, the motivation to decide on actual health care options differed among respondents and was dependent on how they felt  physically and mentally: older people form a heterogeneous group when it comes to preferences on participation in decision-making (differences in culture or personal differences, but we also believe that participation wishes may decline over time as people feel that physical or mental fitness is decreasing). Perceiving a certain level of control in health care was often a matter of adapting mentally, a mechanism that is referred to in the literature as secondary control. |
| Denson LA, et al. Discharge-planning for long-term care needs: the values and priorities of older people, their younger relatives and health professionals. Scand J Caring Sci 2013;27(1): 3-12. | To compare the opinions and values of frail elders living at home, younger relatives and health professionals experienced in discharge-planning, prospectively: before, not after, a long term care decision.  Frail older people,  relatives and  health professionals  Australia | Different stakeholders point out different values and priorities for long-term care after discharge: 1) frail older people give priority to autonomy and protection, 2) relatives to safety finances and the value of living at home, 3) health professionals to safety, then autonomy and personal care.  Culture plays a role in defining these value and priorities | Clear suggestions emerge from these data for improving the discharge-planning experience through theory development, professional training and community education. Ethical care models can specify psychological and physical safety as components of beneficent discharge-planning and acknowledge transitions and relationships. |
| Dick K and Frazier SC. An exploration of nurse practitioner care to homebound frail elders. J Am Acad Nurse Pract 2006;18(7):325-334. | To identify and classify care activities of nurse practitioners (NPs) providing primary care for frail homebound elders and to describe NPs' perceptions of the outcomes of their care activities  Nurses  US | Most care activities were centered under 1. Management of patient health/illness in ambulatory care settings: access to much needed primary care to frail elders that would otherwise not be received. The reported heightened sense of awareness of their patient’s health is consistent with the hypervigilance of NPs working with the elderly. It is also consistent with a model of early recognition of client status changes. Early recognition of changes depends on the therapeutic relationship, which requires continuity of care and trust. 2. Organizational and work-role competencies. The working day of NPs seeing elderly patients in the home is very different from that in the ambulatory care setting. Each patient situation is unique and the NP has to work with, and often build, a different team for each patient.  3. Teaching–coaching function Teaching the caregiver was added as a competency because of the importance that the NPs placed on including the caregiver in all aspects of teaching. Often the family member or other caregiver is providing the hands-on care; other times, the patient has poor memory and providing information to the caregiver is a means to reinforce the teaching as well as provide support to the caregivers.  Although the informants in the current study reported taking care of medical complaints, they agreed that the physical examination takes the least amount of time during the visit. and that the focus on the psychosocial and functional issues complicates the picture. They consult with their collaborating physicians regarding medical issues, but the physicians are also likely to consult with them regarding psychosocial issues. They believe that teaching is effective in the home because (a) the NP sees the actual home situation, (b) there is a constant and trusting relationship, (c) there is repetition of information, and (d) the NP takes time to write the information down and (e) include the caregivers. | NPs deliver expert nursing care by providing compre- hensive social, functional, emotional, and physical assess- ments and by teaching the elder and caregivers how to manage multiple diseases and disabilities. |
| Donlan WT. The meaning of community-based care for frail Mexican American elders. International Social Work 2011;54(3): 388-403. | The rapid increase and the higher rates of disability  and the underutilization of services by Mexican older people suggest the need to illuminate the complex social and cultural context of the experience of frail Mexican-origin frail elders receiving assistance.  Frail older people  US | Deeply held preference for receiving care from family members, expectation of support from family and perceived obligation to provide support. Obligation stemming from love inside the family. Marked problem to see these values in the new generations, US natives. This increases the painful awareness of societal devaluation related to an identity of old and frail. Marianismo (devotion to family and sacrifice for the family) and machismo (challenging physical labour engaged to support the family), with a clear division of roles. Machismo can block the conditions facilitating acceptance of frailty as non-devaluing. Religion and spirituality had two faces: passivity, relying on tradition, caregivers, and religious beliefs useful to support wishful thinking for a miraculous recovery, or the help of God for those who try to help themselves. | Cultural dissonance between generations Expectations from daughters, particularly if living at home and placement of elders in nursing home as failure of family caring. The caregiving burden may place these women at risk for excessive stress with consequences on their own wellbeing and that of their older family members. The solution could be organizing good informal networks among caregivers than offering residential care and other formal services. Practitioners should also be aware that men adhering to rigid gender-role schemas are more vulnerable to low self-esteem and depression when their aging threatens acts upon which the self-identity was founded. These men are more likely to get isolated and use passive strategies of coping, including withdrawal. Practice with these persons should enhance empowerment and use strategies that encourage to be more flexible in exploring activities previously felt as culturally incompatible and which can be shown as self-affirming. Services should be more flexible, like allowing caregivers  to stay overnight in public housing, take care of the initial encounter so that can be as friendly as possible, and get more respectful manners with a population particularly sensitive to this aspect. |
| Ebrahimi Z, et al. Frail elders' experiences with and perceptions of health. Qual Health Res 2012;22(11): 1513-1523. | To explore frail elders' experiences with and perceptions of the phenomenon of health so as to develop a deeper understanding of living with diseases and disorders in old age.  Frail older people  Sweden | Health for frail older people was described in terms of harmony and balance of the salient components of health in their everyday lives. These components included being able to master daily life, the experience of the body working by itself, being happy and satisfied with one’s existence, being validated as a worthy and competent person, and being involved.  These frail elders described the experience of health in relation to its historical context, and in relation to their interests and habits. The experience of harmony and balance was influenced by characteristics inherent in the individual elder and factors in the social environment surrounding him or her. Being in harmony and balance depended on these essential intertwined building blocks, and how they were balanced on the fulcrum. Inner peace and satisfaction were necessary components of the experience of harmony and balance, and these depended on the individual’s insight and understanding of changing life conditions. | The frail elders’ experiences of health in this study were not contrary to other people’s experiences of health. This means that the phenomenon of health is a conceptualized one, because harmony and balance are difficult to measure quantitatively. The study recommends that health and social care staff should pay more attention to frail elders’ everyday lives, and support them in their efforts to balance the building blocks of the phenomenon of health to promote harmony and balance. Medical and social care staff can support frail elders’ experiences of health by focusing on the health balance point in the everyday lives of individual elders. |
| Ebrahimi Z, et al. Health despite frailty: exploring influences on frail older adults' experiences of health. Geriatr Nurs 2013;34(4): 289-294. | To explore and identify influences on frail older adults' experience of health. adults taken from a broader sample from a quantitative study on health.  Frail older people  Sweden | The main theme was identified as feeling assured and capable. The five subthemes related to feeling assured and capable consisted of: managing the unpredictable body (the body was experienced as a barrier to being healthy, as an unpredictable entity that deceived and was no longer able to serve). The older adults with manageable symptoms and disorders were more likely to experience safety and control, thereby experiencing good health, reinforcing a positive outlook, positive thinking and willingness to go on facing further challenges were dependent on awareness of age- and disease-related changes; good mood and willingness to carry on and live despite consistent body hindrances were important driving forces; knowledge and information about resources to compensate for loss of function helped to meet the future; insight, acceptance and adaption shaped willingness and strength to carry on and provided the foundation for a sense of meaning, remaining in familiar surroundings (many preferred to adapt the home environment in order to stay in familiar surroundings, some moved from their homes to accommodate their health conditions so they could continue to manage their everyday lives and having a sense of belonging to the whole), sense of belonging and connection to the whole (social interaction validated a sense of connection to others and the rest of the world, which evolved through contact with others and having someone in their lives that cared about them). | We advocate ongoing assessment and dynamic treatment plans implemented by multiprofessional teams that support older adults’ assuredness in their everyday lives. A multi-professional team is more likely to attend to the whole person in context, facilitate the managing of the unpredictable body, provide opportunities to remain in familiar surroundings so that older adults can manage their everyday lives and have a sense of belonging and connection to the whole. |
| Ekelund C, et al. Self-determination among frail older persons -- a desirable goal older persons' conceptions of self-determination. Quality in Ageing & Older Adults 2014;15(2): 90-101. | To explore older persons' different conceptions of self-determination.  Frail older people  Sweden | Three categories emerged, showing the variations of conception of self-determination as experienced by frail older people: first, self-determination changes throughout life; second, self-determination is being an agent in one’s own life; and third, self-determination is conditional. In summary, while self-determination is changeable throughout life, and older persons want to be their own agents, and struggle to be that, certain conditions must be met to make it possible for them to be able to exercise self-determination. | This paper gives suggestions for 1) supporting and strengthening frail older persons’ self- determination, and indirectly their well-being and health; 2) having a person-centered approach, treat them with dignity and respect ; 3) improving frail patients' health literacy; 4)  make frail patients feel safe and secure in relationships. |
| Ekwall A, et al. Compensating, controlling, resigning and accepting-older person's perception of physical decline. Curr Aging Sci 2012;5(1): 13-18. | To know about how frail older people experience their physical decline and how they adapt to their bodily changes so that the health system can design preventive interventions targeting this group early on in the disability process. The aim of this study was to explore how older people perceive their physical decline.  Frail older people  Sweden | One dimension was the physical decline and its impact on the individual’s physical body and the other was the impact on the body in its environmental context such as the home or the society. The strategies for adapting constituted the two sub-themes, compensating/controlling and accepting/resignation. The strategies were executed both on an intellectual level and practical level.  Controlling and compensating were strategies closely linked. Controlling included various strategies that enhanced feelings of being in charge of the situation, including to keep everyday life as unchanged as possible in relation to how it was before the physical decline. Compensating covered various ways of handling the situation practically when control was lost. Being able to rationalize what had happened or what was happening to the body was being in control of one’s physical decline. Not being in control was revealed in fears of being unable to function and to perform those activities in daily living that were important for the person and part of his/her identity. Losing control by becoming cognitively impaired or having an accident or an acute illness could mean the risk of losing independency. This was described as worse than not being alive at all. Others can help in having control, but this is not negative. When the individual was unable to compensate and stay in control, two strategies were employed: accepting and resigning, a way of restoring control in the new situation, which in turn could be a starting point for compensation in another way. Acceptance was an active way of relating to the fact that some aspects of the physical decline could not be compensated for or controlled and that life was altered forever. Resigning was surrendering and living with the physical decline without accepting it and this was described as sad and hopeless. But there was also a feeling of being forced to take certain medications that had side effects. Awareness that life had changed for the worse. It was also shown in situations when other people took charge of decisions regarding one’s own health or needs. | The gap between the person's possibilities and the actual result reflected the perception of themselves as persons (i.e. thorough, neat and tidy), and their goal was to compensate for this gap in any possible way. For healthcare workers striving to increase physical activity, knowledge about how closely related self image and physical ability are is useful when helping the frail older people. Increasing coping strategies for handling the general life situation may be a useful way of increasing physical activity and making it feel meaningful, despite the person’s frail health situation with limited physical and sometimes psychological resources. |
| Faes MC, et al. Qualitative study on the impact of falling in frail older persons and family caregivers: foundations for an intervention to prevent falls. Aging Ment Health 2010;14(7): 834-842. | To explore the impact of falling for frail community-dwelling older persons with and without cognitive impairments who have experienced a recent fall and their primary family caregivers  Frail older people and caregivers  Netherlands | The impact of falling for frail community-dwelling older persons can be summarized at different level: physical consequences: patients reported physical consequences of their falls, including fractures and minor injuries, such as soft tissue injuries and head wounds; emotional consequences: patients described a constant fear of falling. Patients also described undirected fear, fear of losing independence and negative emotions, such as frustration, anger and disappointment; social consequences: patients recognised that they became (more) dependent on their caregiver after falling. In relation with falling, they observed three coping mechanisms with respect to falling in general: problem-focused coping (actions taken to prevent future falls),  emotion oriented coping (thoughts reflecting acceptance of the fall problem) and avoidance-oriented coping (avoiding certain situations or activities).  All patients ascribed some or all falls to an unknown origin. | A preventing programme should result in more awareness of the risk factors and consequences of falls, of how to walk, to fall and to stand up safely and how to feel more secure. Caregivers' involvement is needed.  Issues of cognitive impairment in any rehabilitation strategies were a concern to carers: "mother isn't teachable". Or they perceived a programme as useless because of their relative's age "Mother is already 80… such a programme would be useless" |
| Fjelltun, AM, et al. Nurses' and carers' appraisals of workload in care of frail elderly awaiting nursing home placement. Scand J Caring Sci 2009;23(1): 57-66. | To describe carers' and nurses' appraisals of workload in care of frail elderly awaiting nursing home (NH) placement.  Nurses and caregivers  Norway | Both similarities and differences between carers’ and nurses’ appraisals of workload in care of frail elderly awaiting NH placement. Either psychological and physical burden was evaluated higher by caregivers than nurses. Carers seemed in despair and expressed stronger feelings of responsibility and worry. Giving care up to 24 hours a day made them more exhausted and isolated, too. Nurses too worried about the elderly. Nurses were frustrated about the lack of NH beds, lack of resources in home health care to do an appropriate work and lack of directions for how to handle situations when elderly refused care. Differences between caregivers and nurses were about use of coercion with nurses more cautious. A common feeling in both was being inadequate.  Three categories were found, namely ‘feeling responsible’ (love, duty and burden of responsibility), 'feeling burdened’ (‘feeling exhausted’, ‘feeling worried’, ‘feeling helpless’, ‘feeling depressed’, ‘feeling isolated’, ‘feeling bitter’ and ‘feeling troubled conscience’), and 'feeling ambivalent’ (about NH placement and about use of coercion). | These results may help guide policy development to address resource allocations to elderly care. Further research is needed to explore life conditions for carers and frail elderly, as well as work conditions for nurses working in home health care. |
| Fjelltun AS, et al. Carers' experiences with overnight respite care. A qualitative study. Nordic Journal of Nursing Research & Clinical Studies / Vård i Norden 2009;29(3): 23-27. | To explore experiences with overnight respite care (ORC) of Norwegian carers who provided care to frail elderly awaiting nursing home placement.  Caregivers  Norway | ‘Being able to continue caregiving because of ORC’, Some carers were able to engage in refreshing activities while the elderly received ORC, ‘Being satisfied with ORC’s quality and/or regularity’. Some elderly could flourish during ORC be brought out of depression. Some carers experienced ORC as supportive for the family as a whole. To accept and have advantages from ORC, it was crucial that ORC was advantageous for both the carers and the elderly in the long run.  Some elderly refused to move to an ORC, and few carers would forcefully move their loved ones. It was crucial to the carers that ORC was flexible and adjusted, meeting both the carers’ and the elderly’s needs. ORC should be experienced as supportive for the family as a whole. To get the intermittent and necessary relief to be able to continue their caregiving, carers had to be assured of the elderly’s safety and well-being. | If ORC was not adjusted to meet the elderly’s needs, some carers felt ORC even worsened their situation. Instead of providing a standard solution, the individual carers’ and the individual elderly’s needs should be considered. |
| Grenier A and Hanley J.  Older women and 'frailty' - Aged, gendered and embodied resistance.  Current Sociology 2007;55: 211-28. | This article draws on complex forms of resistance of older women to the concept of frailty  Frail older people  Canada | Individual Resistance to Being Classified as Frail. On an individual level, older women’s stories elaborate on the material and discursive elements of ‘frailty’, reveal resilience to coercive acts of power and highlight the importance of a sense of self within their life course identity. Older women’s resistance also came in the form of making claims. However, in the shift to risk-based eligibility for services less space is afforded to claims-making and eligibility based on personally defined need for care. Nonetheless, older women emphasize the importance of rights-based entitlements, revealing the extent to which services have shifted away from needs and rights, and the power expressed through organizational practices and concepts such as ‘frailty’.  The flipside of resisting ‘frailty’ is the intentional subversion of the concept to resist social constructions and/or make demands for access, justice and respect. At times, older women subverted negative assumptions using the imposed identity for personal or collective benefit. This subversion is more likely where older women ‘know the rules’ and choose to project themselves as ‘frail’ to gain access to services, manipulate beliefs and/or to have their needs met. However, such subversion within the context of powerful social practices means that older women must negotiate the fine balance between social notions devaluing their bodies and their capacities, the physical realities of change and their self-image. | Their experiences and actions reveal how resistance is different from typical conceptualizations – challenging the form, location and space of resistance. The importance of self and the complex forms of resistance (ranging from rejection to subversion) challenge the understanding of resistance as a direct overt action against an oppressor. Older women’s resistance is more complex than the previous ‘face to face opposition between the powerful and the weak’. Resistance is not only located within the public space, or in the private, but an amalgam of the two. Older women’s resistance may be private as well as public, personal as well as collective, direct and subversive and embodied, as well as conscious and emotional. |
| Gustafsson S, et al. Swedish Health Care Professionals' View of Frailty in Older Persons. Journal of Applied Gerontology 2012;31(5): 622-640. | To elucidate health care professionals' view of frailty in older persons.  Health professionals  Sweden | Being bodily weak and ill, including cognitive functions; being negatively influenced by personal qualities: motivation for engaging in one’s life situation, interest in trying new solutions, other individual values, low expectations in one’s abilities connected to frailty; lacking balance in everyday activities: inactivity or lack of exercise and the opposite, but as a cause of frailty, for instance for partners who have to take care of the other partner; dependency on others if they need help for everyday activities; not being considered important: not heard, older persons are not needed and consequently not being in demand as persons (not being considered in societal development, like being asked for opinions in the design of retirement homes leads to frailty); hindrances in the home, not adequately equipped, but also not served by public means or with no community services available, difficulty in using new technologies, having no personal economic asset, and also all that does not really help the older person; having an inadequate social network: absence of relatives and weak social network, no possibility to share and no support.  Frailty has only relatively to do with age. Dynamic of frailty with a continuum of stages. | Dynamic of frailty with a continuum of stages means that it is possible to identify a prefrail state, probably more suitable for interventions.  A revision of the material used in health consultations for older persons to encompass all identified dimensions would be useful. |
| Hjaltadottir I and Gustafsdottir M. Quality of life in nursing homes: perception of physically frail elderly residents. Scand J Caring Sci 2007;21(1): 48-55. | To disclose the characteristics of quality of life as perceived by physically frail but lucid elderly people living in nursing homes to increase the understanding of the phenomenon of quality of life in this setting.  Frail older people  Iceland | QoL is a multidimensional phenomenon. The four themes identified are: securing the body (the NH can do that), seeking solace (more problematic, in particular given the presence of very demented people having disturbing behavior), preparing for death (it can be done), affirming the self (links with family, appreciation of past life, learning things, not wasting time: NH life should do better from this point of view).  Securing the body included ‘Being safe’; ‘Being cared for’; and ‘Maintaining the body’. Seeking solace: ‘Feeling at home’; ‘Habitual way of being’; ‘Overstepping the confinement'.  Preparing for departure: a time of transition. Affirmation of self: ‘Being recognized as a person’; ‘Family relations’; ‘The lived past’; and ‘Doing meaningful things’. | Some aspects of quality of life depicted in this study need to be addressed at the structural and organizational level of the nursing home, such as the safety of the residents and availability of single rooms. Other factors need to be addressed at the individual level, for example with care that is adapted to individual needs and wishes, while considering other factors relative to the person’s past life, disposition and personality, as well as his mental and physical capacity. Caring for frail residents in nursing homes needs to be approached with professional knowledge and skills with regard to the person’s situation in life and his/her concerns. |
| Horder HM, et al. Self-respect through ability to keep fear of frailty at a distance: successful ageing from the perspective of community-dwelling older people. Int J Qual Stud Health Well-being 2013;8: 20194. | To explore successful ageing from the perspective of community-dwelling older people (24 persons aged 77-90 years)  Frail older people  Sweden | An overarching theme was formulated as ‘‘self-respect through ability to keep fear of frailty at a distance’’. This embraced the content of four categories: ‘‘having sufficient bodily resources for security and opportunities’’, ‘‘structures that promote security and opportunities’’, ‘‘feeling valuable in relation to the outside world’’, and ‘‘choosing gratitude instead of worries’’. More in particular: Satisfaction with one’s financial situation, Security and opportunities in the closest context, The health and well-being of close relatives and friends, Choosing gratitude for not being as bad as others who are in a worse situation, Denying difficulties, Accepting things you cannot change.  Ageing seems to be a dynamic process rather than a static structure and might therefore be susceptible to actions. Self-respect is linked to recognition of our place in society, dealing with our sense of autonomy, mastery, and our place in society. To preserve one’s self-respect, too much focus on oneself was not desirable and the ability and conditions for keeping involved in other persons or interests were emphasized. Attention has been drawn to this struggle to preserve one’s identity according to a developmental  theory, a life-course perspective on identity seems needed to reach an ability to adapt to losses and stressors in old age. External factors, especially economy, were of importance to avoid worries and enable involvement. | Paying attention to and respecting worries regarding an increasing vulnerability, while at the same time focusing on older persons’ abilities and strategies for preserving their self respect, can lead to better ways of promoting successful ageing in those still living in the community. This highlights the importance of societal attitudes towards ageing, which are reflected in older persons. |
| Kita M and K Ito. The caregiving process of the family unit caring for a frail older family member at home: a grounded theory study. Int J Older People Nurs 2013;8(2): 149-158. | To explore the caregiving process of family units caring for a frail older family member at home.  Caregivers  Japan | Routinisation as incorporation of changes, against confusion and Minimisation of competing need share in relationship so that four possible situations are created: confusion, fluctuating disharmony, stable disharmony, stable harmony. Confusion: no routinisation of daily life. Family members performed at-home care in a state of confusion. No consciousness of the difficulties accompanying the restriction of activities and interactions arising from the at-home care. Family members did not feel the need for outside services and there was a tendency to refuse outside services even when suggested or recommended by a professional care provider. Fluctuation: Daily life had not become routinised, but family members felt that difficulties would arise with at-home care if the situation continued unchanged. Family members strove towards eliminating the restrictions imposed by at-home care and actively attempted to seek out and introduce outside services. Stable disharmony: Daily life had become routinised and major competition of needs existed among the internal family members, resulting in difficulties with at-home care. Although a growing sense that the situation could not continue, things were routinised; therefore, a strong trigger or outside force was needed to bring about a change, such as the introduction of new services. Stable harmony: The needs of each family member were sufficiently fulfilled and the family’s daily life had become routinised. This state provided the optimal level of ease of lifestyle. This is the outcome of coping strategies in the family or inability to communicate and balance needs. The optimal status for the caregiving family could be explained by the highest level of routinisation of daily life and the lowest level of competing needs within a family. The degrees of these two categories were found to be changed by several conditions and family strategies.  To eliminate confusion and unpleasantness, family members made continuous efforts towards routinisation of daily family life. One approach was to incorporate risk management strategies into daily life (to anticipate change in the illness state of the older member or problems with medical equipment, to find daily routines that incorporated plans for preventing any changes or problems, to acquire patterns to manage emergency situations). | Families have the potential for self-regulation and self organisation and improving the various family’s coping strategies is a potentially very powerful form of intervention. Nurses should support or enhance family strategies for routinisation of daily life and minimisation of competing needs within the family. They should support the family to regulate their competing needs by listening empathically so that family members can reconfirm the meaning and importance of the care they are providing, provide opportunities for family to share care experiences, and offer information about prognosis and predictability for the future. |
| Koenig TL. From the woman's viewpoint: Ethical dilemmas confronted by women as informal caregivers of frail elders.  Families in Society-the Journal of Contemporary Human Services 2004;85: 236-42. | Explored women's ethical decision-making in caring for a frail elder.  Caregivers  US | Anger, guilt, sadness, and fear are some of the feelings that caregivers described as part of dealing with or resolving ethical dilemmas. Often, feelings arose before, during, and after the ethical decision-making process. The occurrence of feelings in the decision-making process reflected the ongoing nature of ethical dilemmas that are initially struggled with and often continue to be dealt with over time. | Findings are integrated into an ethical decision-making model that includes types of ethical dilemmas (e.g., protection of life vs. autonomy); feelings that permeate decision making (e.g. , fear); processes for addressing ethical dilemmas (e.g. , family collaboration); and supportive services. Implications include (a) expansion of services to meet caregivers' ongoing needs, (b) research that acknowledges multiple decision-making components, and (c) the use of caregiving vignettes in the classroom. |
| Kristensson J, et al. Frail older adult's experiences of receiving health care and social services. J Gerontol Nurs 2010;36(10): 20-28; quiz 30-21. | To explore frail older adults' overall experience of receiving health care and/or social services.  Frail older people  Sweden | The  main  category  revealed  the  older  adults’  experience  of  receiving health  care  and/or  social  services  as having  power  or  being  powerless. Subcategories were:  autonomous  or  without control  in relation  to  the  health  care  and/or  social  services  system,  being  confirmed  or  violated  in relation  to caregivers,  and  paradoxes  in health care  or  social  services.  Knowledge that the  hospital was  obliged  arrange interventions enhanced the feeling of  security  and  the understanding  that  care would  be  provided  as expected. However, there were  also feelings that these rights were at risk and had to be struggled for.  The  participants’  ongoing  relations with health care and/or social services was largely concerned with  power and the struggle to obtain or  remain in power, which in turn had implications  for  their encounters  with  staff  and  for  their satisfaction with the care and services provided and with their entire situation in  terms  of  self-esteem. The distribution of power as an extrapersonal phenomenon was revealed in the participants’ relationship to the system. In situations when the power was distributed in  favor of  the older adult, they  spoke about  care  or  services  as  an  everyday,  unproblematic events. Being powerless as an extrapersonal phenomenon was seen in a lack of continuity and being in the hands of the organization. | The issue of power involves an extrapersonal  perspective  (the encounter  between  the  individual and  the  organization);  an interpersonal  perspective  (the encounter  between  the  individual and the caregivers); and an intrapersonal  perspective  (processes within the  individual). If health  care  and/or  social  services as  systems  function  properly,  this contributes  to  positive  encounters, confidence, and  satisfaction,  thereby empowering the person. Fragmentation among care providers has been identified as a problem in care and services for older adults. Empowering strategies on an extrapersonal level could perhaps be introduced by providing  information about  the  system and  its structures and by confirming that the older adult is a part of the decision-making processes and ensuring the person is aware of his or her rights in relation to the system. When needs are not met, feelings of being powerless and of being less worthy are evoked. The encounter with the nurses  and other health  care professionals stood out as vitally important related to the feeling of being in power or powerless.  It is important for nurses and other health care professionals to strive to achieve a high level of integrated care and interpersonal  continuity,  as  this may  contribute  to relationship-based care and feelings of  security, participation, and control.  When professionals at all levels provide health care or  social  services  through  a  variety of agencies, they need to be aware of the risk of violating frail older adults’ feelings that they are in charge of their life situation. This requires coordination,  information,  accessibility, and continuity on both organizational and interpersonal  levels.  A case manager with gerontological nursing competence might  be useful  in this process. |
| Levesque L, et al. A partnership approach to service needs assessment with family caregivers of an aging relative living at home: a qualitative analysis of the experiences of caregivers and practitioners. Int J Nurs Stud 2010;47: 876-87. | T to explore the experiences of caregivers and practitioners who took part in a field test of the Family Caregivers Support Agreement (FCSA) tool, designed to facilitate partnerships between caregivers and practitioners so that needs assessment and subsequent support services are negotiated and agreed so as to meet caregiver expectations.  Caregivers, nurses, social workers  Canada | The main strategies that practitioners use to engage caregivers as partners in the assessment process were sensitive listening to their concerns and use of enabling questions to foster their abilities to ﬁnd creative solutions to the challenges they face. The caregivers described experiencing a climate of trust that allowed them to express their concerns, to reﬂect upon their situation and to participate in the development of an action plan for their support. This strategy allows caregivers the chance to vent their emotions, which has a cathartic and potentially therapeutic value. If they are not given the opportunity to be heard, caregivers often feel misunderstood and a relationship of distrust can develop, thus undermining any potential partnership. Enabling questions subtly led caregivers to reﬂect upon their situation in order to gain a more accurate view of their needs, and to highlight their ability to ﬁnd creative solutions tailored to their needs. This strategy seems to foster reﬂexive learning in caregivers, which is empowering in that it leads to a deeper understanding of one’s situation.  Caregivers and practitioners took an equal part in the process started by the tool and in so doing came to know more about both their roles and their perceptions. The partnership process also provides insights into why some caregivers are reluctant to seek help and of how to overcome this. Emphasizing caregivers’ strengths and their ability to ﬁnd solutions helped caregivers view help-seeking not as an indication of failure but rather as a resource to allow them to go on taking care of their relative. | Adequate assessment of caregiver needs must lie at the heart of an appropriate service response in which caregivers are viewed as genuine partners with support needs of their own if they are to enjoy a better quality of life. The implementation of a partnership approach would force political and institutional authorities to take measures to ensure that nurses and other health practitioners are allowed the requisite time to apply the tool. If such beneﬁts are to be realised it must be recognized that practitioners themselves will need appropriate training and clinical supervision in order to reinforce their capacity to respond to the emotional needs of caregivers. |
| Lindhardt T, et al. Nurses' experience of collaboration with relatives of frail elderly patients in acute hospital wards: a qualitative study.  Int J Nurs Stud 2008;45: 668-681. | To illuminate nurses' experience of collaboration with relatives of frail elderly patients in acute hospital wards, and of the barriers and promoters for collaboration.  Nurses  Denmark | The main finding is the contradiction between nurses' professional ideals and knowledge and their own perception of relatives as demanding and often people to avoid in everyday practice. In addition, while they acknowledged that engagement with relatives should be planned and part of care, in practice encounters were often just coincidental. Moral conflict and "escape-avoidance" was discussed.  The practical, rather than the ideal actually governed their conduct, and led to tension. However, there wasn't a lack of empathy, just a lack of actual structure. None collected information about the relatives' potential to take care of the patient when discharged, only one had elicited any knowledge about how a patient had coped at home before hospitalisation. Discharge planning was the most likely time for relative involvement. Lack of time was mentioned but clarified as a lack of structure and prioritising, but also feeling unprepared for the task. Social needs and collaboration with relatives was seen as a low priority. Competence, structure and being proactive was seen as the ideal for all parties. | Nurses seemed to be victims of organisational and societal, macro-level value systems that do not support collaborative efforts. Micro level barriers, such as structures and ethos of particular wards were seen as more surmountable, e.g. the role of the nurse leader/manager as role models and promoters. Promoting continuity of care within wards was a recommendation, as was increasing nurses communication skills. The reflection involved in doing the interviews was recognised as useful by the nurses, so recommended as well. |
| McGeorge SJ. Unravelling the differences between complexity and frailty in old age: findings from a constructivist grounded theory study. J Psychiatr Ment Health Nurs 2011;18(1): 67-73. | Tto explore findings from a study into how mental health nurses who work with older people construct and operationalize the concept of 'age-related complexity'.. This paper addresses the relationship between frailty and complexity, which was identified as a theme within the category 'dynamic complexity'.  Nurses  UK | Frailty is exclusively used to describe physical states while complexity is a more encompassing term that has resonance and relevance in mental health services. Frailty is described principally in relation to older people’s physical state, whereas complexity is a consequence of the interaction of needs across a number of areas. Frailty and complexity share some characteristics; both are seen as common in older people, both may require complicated nursing care, both tend to be associated with involvement of multiple services or agencies, but there is a greater number of differences. Nurses did not think about frailty in relation to mental health. There is ‘no going back’ from frailty, and the identification of an older person as ‘frail’ signals a continual decline in functioning and health status, sometimes represented on a continuum and placed between independence and pre-death. | The findings from this study suggest that complexity and frailty should be recognized as distinct concepts and states, raising the possibility that frail older people and older people with age-related complexity may require different approaches and nursing skills, and further research in this area is important. Furthermore, the study of complexity in old age offers the opportunity to move away from preoccupation with frailty, together with its potential to medicalize and problematize old age, and consider a more optimistic approach to recognizing older people’s differences, needs and potential. |
| Modig S, et al. Frail elderly patients' experiences of information on medication. A qualitative study. BMC Geriatr 2012;12: 46. | To explore frail elderly patients' experiences of receiving information about their medications and their views on how the information should best be given  Frail older people  Sweden | Two main categories: confortable with information and insicure with information. Confortable is conditional to trust in physician or medication, enough information from the prescriber, possibilty to rely on yourself for information. Insicure if there was anxiety, if medical care is poor and visits too quick, and information hardly given.  Knowledge/taking control/security. Security is linked to trust and confidence in the doctor or medications. Repeated information was appreciated. Insecurity was linked to distrust, disappointment and insufficient information, lack of availability when information is needed about possible side effects. | Confidence means that elderly people often do not take active part in decision-making. |
| Nicholson C, et al. Living on the margin: understanding the experience of living and dying with frailty in old age. Soc Sci Med 2012;75(8): 1426-1432. | Understanding of the experience and challenges of living and dying with frailty in older age  Frail older people  UK | Three main themes arose from the analysis: 1-The dynamics of physical and psychosocial frailty, which details the persistent state of uncertainty and loss experienced as a result of progressive physical and psychosocial changes; 2- Sustaining connections within the home presents the work that older people engaged in to remain anchored within the imbalances of their frailty, 3-Connecting with death and dying addresses these older people’s work of acknowledging and communicating finitude within frailty.  Visible markers of functional limitations and the increasing social losses of old age bring finitude to the fore. To retain anchorage in this state of imbalance, frail elders work actively to develop and sustain connections to their physical environment, routines and social networks. This experience can be conceptualised as persistent liminality; a state of imbalance “betwixt and between” active living and clinically recognised dying. This condition is often perceived as having a "marginal and isolation status" | Frailty is a persistent liminal state. Participants’ narratives capture the feelings of uncertainty and loss experienced with progressive physical and psychosocial changes and an increased awareness of finitude. Moreover older people’s stories revealed considerable capacity to create daily routinised practices to anchor themselves and sustain connections within their imbalance. However the study also reveals the problematic nature of finding shared meanings between older people and health and social care professionals within the continual and shifting state of frailty.  Current theoretical understandings and services for the frail old should favor more  the persons in the context of their identity and their relationships, rather than physical body. Uncertain work of maintaining identity through frailty. |
| Nicholson C, et al. The experience of living at home with frailty in old age: a psychosocial qualitative study. Int J Nurs Stud 2013;50(9): 1172-1179. | Tto understand the experience over time of home-dwelling older people deemed frail, in order to enhance the evidence base for person-centred approaches to frail elder care  Frail older people  UK | Frailty is conceived as a state of imbalance in which some connections are lost and new ones are created. Loss of physical capacity, social status, friends and family, autonomy. The loss of autonomy is linked to self-identity, maybe caused by the contact with services. Strategies to reconnect them to their bodies and daily lives, often grounded in the tasks of eating. Much effort in maintaining and sustaining their place in their immediate world: daily routine, continuity of social relationships. Connected to this is the risk of breaking these ritualized patterns, ever present in the uncertainty of changing physical and social capacity.  This study argues that focus on the work of holding together loss and capacity as part of a person centred approach to care for frail older people is necessary. | To look for a balance between autonomy and dependence and changing role. Relational creativity underpinned by the capacity to connect to their changing circumstances and adapt in the ambiguity and discinnections of being frail. Experiencing frailty requires a delicate balance succesfully to integrate previous, present and future realities. Connection over time. |
| Puts MT, et al. The meaning of frailty according to Dutch older frail and non-frail persons. Journal of Aging Studies 2009;23(4): 258-266. | To describe the meaning of quality of life from the perspective of frail and non-frail older community dwelling persons.  Frail and non-frail older people  Netherlands | Three dimensions in frailty: physical functioning, psychological/cognitive functioning and social functioning. State of multiple health problems combined with other problems, like anxiety and isolation. Frail respondents more often stated that fraiialty is a process which cannot be controlled. Non-frail ones mostly described other persons as frail and say frailty can be prevented by taking actions.  Different between men nd women in the importance of themes. Men emphasize physical dimesnion and women psychological. | For older persons frailty in addition to specific physicocial components also denotes psychological and social problems. Emotional and social domains should be included when assessing frailty. |
| Puts MT, et al. What does quality of life mean to older frail and non-frail community-dwelling adults in the Netherlands? Qual Life Res 2007;16(2): 263-277. | To explore the meaning of quality of life to older frail and non-frail persons living in the community.  Frail older people  Netherlands | Health, well-being and social contacts were considered the more important factors in quality of life.  Social contacts were more critical for frail persons, and also more important. Financial possibilities were also involved. The perception of the neighborhood, the feelings of safety in the neighborhood, especially after dark, were mentioned as important. Non-frail respondents mentioned health as most important and necessary to enjoy life, and thus well-being. For the frail persons, social contacts were most important and described as necessary to well-being. | Existing instruments for measuring quality of life may not be valid for older persons since they do not capture all the themes mentioned by older persons. |
| Robben S, et al. Preferences for receiving information among frail older adults and their informal caregivers: a qualitative study. Fam Pract 2012;29(6): 742-747. | To explore the experiences of frail older people and informal caregivers with receiving information from health care professionals as well as their preferences for receiving information.  Frail older people and caregivers  Netherlands | Frail older people and informal caregivers varied in their information needs and discussed both positive and negative experiences with receiving information. They preferred receiving verbal information from their physician during the consultation; yet would appreciate receiving brief, clearly written information leaflets in addition. They employed several strategies to enhance the information provided, i.e. advocacy,preparing for a consultation and searching their own information. Contextual factors for receiving information, such as having enough time and having a good relationship with professionals involved, were considered of great importance. | Even if the information provided would meet all their preferences, this would be of limited significance if not provided within the context of an ongoing trusting relationship with a professional, such as a GP or practice nurse, who genuinely cared for them. |
| Roland KP, et al. Exploring Frailty: Community Physical and Occupational Therapists' Perspectives. Physical & Occupational Therapy in Geriatrics 2011;29(4): 270-286. | To explore therapists' perspectives on frailty, and develop a definition of how they view and manage frailty in their practice.  Occupational therapists  Canada | Poor physical fitness was the most common physical frailty characteristic and mostly contributed to decreased functional ability and increased risk for falls. Isolation was the primary psychosocial characteristic and it was related to a client’s physical fitness, general health, medication, capacity to ask for help. Isolation often resulted in a decreased social network and depression due to client’s inability to access their community; it can be less evident if a client depends on family caregivers, social network (“a family buzzing around them”), or has financial resources. Therapists recognized that a combination of declining physical, cognitive, and social characteristics contribute to frailty, but it took a number of visits to identify all these. Therapists’ image of frailty included a complicated medical history: a combination of conditions shifted the client to increased severity. Therapists often associated frailty with a multi-system failure in a complex client. Treatment plan: often it was not based around a universal definition, but rather driven by the client’s individual expression of frailty. Therapists expressed difficulty in determining the difference between pre-frail and frail across the frailty spectrum. They explained they just know it is frailty in practice and there were no treatment algorithms to follow. There was an instinctual nature to their definition, and they were able to adjust their image to fit different people. Observation of clients in their home environment was an assessment used with both pre-frail and frail clients. The therapists thought that implementing exercise programs in a group setting in the community held important advantages over home-based exercise programs, improved motivation, social connections, and are easier to monitor progress. This was especially endorsed for pre-frail adults because therapists thought that enrollment in community programs strengthened their clients’ participation and ability to improve both socially and physically. Adult day programs were recommended for frail clients with cognitive challenges, but to enroll frail older adults in community classes can be difficult due to withdrawal, fear, or immobility. | Therapists’ perceptions of frailty align with current definitions available in the literature. Therapists emphasized declining capacity, physical function, cognitive ability, support networks, complex medical histories, multi-system failures and stressors leading to a tipping point. The personal perspectives of these therapists did not include weight loss as a key characteristic of frailty in their clients. Instead, therapists focused on the impact of physical inability, impaired decision-making skills, lack of motivation, and cognitive incapacity on a person’s social networks and ability to safely access and engage in their community. |
| Rush KL, et al. Older adults' perceptions of weakness and ageing. Int J Older People Nurs 2013;8(1): 1-9. | To understand the meaning of weakness for older adults' and their perceptions of its association with ageing.  Frail older people  Canada | Themes were "The ambiguity of weakness" which refelcted uncertainty of what the question meant, but gave clearer, but contradictory views of it as associated with ageing - as negatively associated with ageing, an accepted part of ageing, unrelated to ageing, and also ageing seen as weakness (with people saying they didn't want to "appear old". Outward and inward causes were given - (i) deficits and limitations in the body or the mind, inability to meet expectations "can't" (ii) self-pity, loss of confidence, frustration, passivity, inability to cope, persevere or fight, lost the will/quit. Attributions of the cause of weakness (i) or (ii) influenced the degree of negativity with which it was regarded.  The "dreaded endpoint" of weakness was discussed - "I don't want to be that way but am getting a bit more that way, I must admit". - the tension between the now and the not yet. Motivating self talk and personal control emphasised, as was achieving balance, such as modifying activities. (SOC again). Resilience, they felt, had more to do with personal strength, not physical capabilities. | The authors point to the "turning inward" point as a potential transition point that should receive focus in further research or intervention, suggesting it was critical but reversible. Enhancing this psychosocial coping perspective was seen as important and often missed by healthcare or social care practitioners. |
| Sarvimaki A and Stenbock-Hult B. The meaning of vulnerability to older persons. Nurs Ethics 2014. | To illuminate the meaning of vulnerability.  Frail older people  Finland | The  meaning of vulnerability to older persons involves physical, mental and social losses that made life more limited and uncertain. The results support previous research describing ageing in terms of frailness. The results also showed that vulnerability meant being unprotected or in need of protection. The interviewees emphasized the role of self-protection, thus relying more on individual assets than family ties and other social networks.  Six themes: being easily harmed, becoming an old person (losses, giving up things, with consequent uncertainty), reactions when being violated and hurt (anger, sadness, disappointment, dwelling), protection (from the others but also being able to protect themselves) and vulnerabilty as a strength (to learn to cope with hurt and feeling pain). | Old people’s frailness often means that their human dignity also becomes vulnerable; they feel depersonalized, excluded and de-valued. In order to protect the older person’s human dignity, the nurse needs to be morally sensitive. Since the core of ethics in the care of older persons lies in the encounter and relationship between the nurse and the vulnerable older person, nurses need to recognize the deep sense of vulnerability associated with old age. Life in old age involves losses, limitations and uncertainty that require special sensitivity of the nurse in order not to harm the person. |
| Skymne C, et al. Getting used to assistive devices: ambivalent experiences by frail elderly persons. Scand J Occup Ther 2012;19(2): 194-203. | To learn how frail elderly people experienced becoming assistive device users and how assistive devices affected their independence in daily activities.  Frail older people  Sweden | The attitude towards assistive devices includes two themes: confidence in knowledge/experience and getting used to assistive devices which are combined as parts of a pendulum. The first is the base because is a prerequisite to be ready to use assist. dev., and the second moves between five categories which contains two opposites: transient/permanent needs, creates opportunities/limitations, physical environment facilitates/complicates, raises concerns/security, social environ encourages/restricts. There are several distinct or opposing meanings: for the relatives the ass. dev. had relatives become overprotective and limited daily activities. Others see the person as "crippled", rather than more capable. | The experience is basically ambivalent. "Place of integration" as process of action that addresses problematic aspects of a situation, remakes it and brings harmony to an ongoing transaction, reconstructing and attributing new meanings. The pendulum means that the more knowledge and confidence you have, the less the pendulum swings.  There is also an empowerment issue the authors see in the efforts needed to involve in the prescription process and in the balance of people's beliefs in their own competence and their trust in the expert. |
| Stockwell-Smith G, et al. Why carers of frail older people are not using available respite services: an Australian study. J Clin Nurs 2010;19(13-14): 2057-2064. | To explore the limiting and motivating factors that influence carers' use of respite services and the ability of currently available respite services to meet the needs of carers of frail older people  Caregivers  Australia | Participants located the socially defined physical care if the care recipient as central to their role. This can be a main reason for refusal of respite services  - they usurp the caregivers' role.  Three themes: Commitment, where the carers shared information on their caring role that embodied reciprocity (I shall give what I had one received or will receive, but also isolation encompassed by the role), role definition (intermediary between person and services, other points not clear) and role frustration (difficulties in everyday care, often caused by care recipient's behavior, but also bureaucracy needed to access basic rights); needing help, as experiences that elicited mistrust, a lack of confidence in services and resulted in fear and resistance; support as informal networking, misinformation, lack of knowledge of community services. | "It was evident that they were disempowered". For caregivers difficult to decipher and negotiate services, frustration in their transactions with service providers when shared care is required. There is a clash of cultures and expectations is a reason to reject formal support. At least communicate: need for information is not related to lack of information, rather to difficulty in understanding the information provided... a common language may be achieved to communicate more effectively. Access at entry: entry criteria are a mechanism to control and exclude. |
| Teixeira IN. [The perception of health professionals of two definitions of frailty in elderly people]. Cien Saude Colet 2008;13(4): 1181-1188. | To understand the perception of health professionals of two definitions of frailty in elderly people  Health professionals  Brazil | Health professionals did not agree with an unidimensional conception of frailty. Six central ideas emerged from the discourses regarding the definition of frailty: 1) unidimensional (Fried) and multidimensional (Bergman) definitions are complementary; 2) there is no potential for prevention; 3) defining frailty is a complex task; 4) what is the concept of frailty? 5) the multidimensional definition is complete; 6) frailty is a state that can last for life. Three tentative definitions of frailty could be constructed: 1) clinical syndrome of reduced reserve and resistance to stressors, presence of several pathologies us of medication and functional and cognitive dependence; 2) interaction of different factors causing decrease in the reserve and increase in vulnerability; 3) decrease in the motor and physiological activity, not necessarily with an apparent cause leading to increased morbidity and mortality.  Fried's definition was considered excessively biological, and inadequate for describing frailty, with emphasis in vulnerability but no determining factors, and not providing definitions for reserve or resistance. Bergman's definition was considered more close to reality, but biased towards a extremely positive view of aging and not taking into account the potential irreversibility of vulnerability. | Difficulties for defining frailty indicates the need of a consensus between professionals. |
| Themessl-Huber M, et al. Frail older people's experiences and use of health and social care services. J Nurs Manag 2007;15(2): 222-229. | To highlight older people's experiences and expectations of services and the consequences for service provision, service development and research.  Frail older people  UK | The older people generally appreciated the care provided by services but also reported feeling uncomfortable in dealing with them. Frail older people are high users of services but claim that services are not responsive to their main concerns: meeting individual needs, maximizing independence and helping to live fulfilled lives. Older people also grew frustrated if services failed to align care provision with either their tried and tested personal routines or the dynamic nature of their needs. They are aware of their needs and abilities and they expect the same of service providers.  They were ambivalent with respect to the process of being admitted to hospital as an emergency. Some were reluctant to contact formal services and in some instances they were opposed to being admitted, but, once in hospital they praised the quality of intramural services and perceived their admission as having been unavoidable. They prefer services that focus on tertiary prevention. In other words, this group of older people would prefer health and social care services to focus efforts on the care of their already established health issues, minimize detrimental consequences and diminish age-related complications. They prefer a service that supports and boosts their capacities, capabilities and social networks and a service that makes them feel safe while remaining inconspicuous when not needed and that ensures easily accessible help in emergency situations. | A key task for community-based services is to promote services that support older people’s independence, address the under-utilization of services and enable older people to participate in familiar and everyday social routines. The older people in this study have stated that services are not yet sufficiently flexible, do not yet involve older people enough and do not adapt care provision to individual circumstances and preferences, including being admitted to hospital. |
| Tutton EM. Patient participation on a ward for frail older people. J Adv Nurs 2005;50(2): 143-152. | To explore the meaning of participation for older people in hospital and their health care workers and ways in which staff can enhance patient participation in their care  Health professionals and frail older people  UK | Participation depends on facilitation style and facilitation strategies. Partnership is involved as made of respect, trust and negotiation. This is a dynamic process of communication, evaluation and change.  Understanding the person as awareness of the personal history, connecting to the person, understanding what of the illness makes her like that, to identify opportunities for change. Emotional work encompassed accepting the other, using emotion in a positive way and dealing with strong emotion. Coping involved staff controlling their feelings and acting in a way that focused on patients and their needs. If this was not successfully achieved, staff could reduce patients' opportunities for participation. | Facilitation of participation needs reflection and control in staff. The desire of patients to cope with institutional life is the desire to cope with uncertainty of hospital life, not the inability to discern about their care. Both patients and staff undertook emotional work. Both learnt to accept each other and patients learnt to accept dependency and hospital life. However, there was tension between accepting dependency on others and maintaining a sense of autonomy. |
| van Kempen JA, et al. Home visits for frail older people: a qualitative study on the needs and preferences of frail older people and their informal caregivers. Br J Gen Pract 2012;62(601): e554-560. | To explore the views and needs of community-dwelling frail older people concerning home visits  Frail older people  Netherlands | Most participants would like to receive home visits. This would restore the type of care they receive "before" when there were less time constraints and professionals had a less impersonal attitude.  They do not want "to bother" the professional, in particular GP. Home visits contribute to trust and attention ("You know someone pays attention to you"). But also scepticism, some could not imagine the GP to have time for home visits.  Some people thought that home visits were useful for very frail and in need patients. Trust depends on continuity of professional. Home visits would improve patent-professional relationship. Trust is higher when the professional is known. Home visits show attention, and this increases trust in the GP. Home visits can improve attention to the psychosocial aspects. | Home visits are appreciated for reasons different than the actual purpose, which focus on prevention of health problems.  The study can explain why the effectiveness of home visits for prevention remains unclear. Patients should be involved in developing home visiting programmes. |
| Walker R, et al. How older people cope with frailty within the context of transition care in Australia: implications for improving service delivery. Health Soc Care Community 2015;23(2): 216-224. | Examines how older people cope with frailty within the context of a dedicated transition care programme and discusses implications for improving service delivery  Frail older people  Australia | Overall, this research highlighted that benefits associated with transition care can be undermined by fragmentation in service delivery, loss of control and uncertainties around future support.  Three key themes: ‘a new definition of recovery’, ‘complexities of control’ and ‘the disempowering system’. Despite describing many positive aspects of the programme, including meeting personal milestones and a renewed sense of independence, participants recognised that they were unlikely to regain their previous level of functioning. For some, this was exacerbated by lacking control over the transition care process while adapting to their new level of frailty. Development involves both growth and decline. Participants could be seen to experience a number of important developmental ‘gains’ from the programme, despite considerable physical ‘losses’ in terms of function and independence. These included being able to remain living at home, some restoration of independence and not having to rely on family support where this was not available, nor preferred. Unsurprisingly, some participants seemed to experience fewer ‘gains’ and their journey encompassed more noticeable ‘losses’ than others. This could potentially be exacerbated when there was a lack of control in terms of either involvement in decision-making around care needs or lack of continuity of care. | This research highlights that good-quality, client-centred, aged care does not mean the development of a homogeneous client response, but one which is individualised and targeted to the needs of each individual’s changing context. Listening to clients and their families and remaining cognisant of their changing needs over time. |
| Wallin M, et al. Physiotherapists' accounts of their clients in geriatric inpatient rehabilitation. Scand J Caring Sci 2008;22(4): 543-550. | To explore and describe the consequences of an acute hip fracture as experienced by home-dwelling elderly people shortly after discharge from hospital.  Physiotherapists  Sweden | Two accounts emerged with regard to rehabilitation with older frail adults: older adults as recipients of a treatment (a one-way flow of interactions), and as partners in an exercise intervention to support their everyday living at home (two-way flow of interaction). In the first account there are two dimensions: focus on physical impairment (conceived apart from the person and sometimes other parts of the body, and from the context) and focus on social needs. Physioth. can describe the encounter with the client as client-centred, but at the same time positioned clients as passive recipients of comfort treatments. Clients were anyway passive recipients. In the two-way modality, the emphasis is more on the individual's resources and on a view of the physical environment. In the one-way flow modality, older adults' domestic context was seldom considered in the therapeutic approach. On the other hand, over-emphasis on social needs also implied passive treatments. | This is really the view of Physiothearapsits' experience say that passivity was the most prevalent characteristics. The physiotherapists did not challenge clients' unwillingness to involve themselves in traditional physiotherapy, and this was constructed as a matter beyond professional influence. Ageism perpetuates negative images and they are seen as dependent, helpless and powerless. |
| Zidén L, et al. A life-breaking event: early experiences of the consequences of a hip fracture for elderly people. Clin Rehabil 2008;22(9): 801-811. | To explore and describe the consequences of an acute hip fracture as experienced by home-dwelling elderly people shortly after discharge from hospital.  Frail older people  Sweden | The subjects described a new experience of physical limitations and eroded independence. Life had become narrower in physical, psychological and social terms. They felt dependent upon others and trapped at home, and all also expressed feelings of insufficiency, related to a limited capacity to move, and lost confidence in their bodies. Trivial daily activities, such as washing clothes, walking stairs or shopping, became difficult. The subjects experienced these consequences as unexpected, and they had an impact on their self-confidence. The feeling of bodily betrayal, not being able to trust their own bodies, was accompanied by a growing feeling of mistrust of their own physical ability, and they regarded their bodies as more fragile and unreliable. Early experienced consequences of a hip fracture were multidimensional and involved dramatic and extensive changes in the life situation, including existential thoughts and reappraisal of the remaining years of life. | To deepen the health care professional’s understanding that the patients’ experiences extend beyond the actual injury. One key question is how far the responsibility of the health care system should go. Does it suffice to take care of the acute medical and rehabilitation needs and leave the rest to the individuals themselves to arrange? Arranging health care and rehabilitation chains in order to link together different health care organisations should be given priority. |
